# The biocontrol agent *Streptomyces rimosus* subsp. *rimosus* tempers shifts in the wheat spicosphere microbiome induced by Fusarium Head Blight

**DOI:** 10.3389/fpls.2025.1540242

**Published:** 2025-02-20

**Authors:** Larissa De Troyer, Kris Audenaert, Sarah Ommeslag, Jane Debode, Leen De Gelder, Noémie De Zutter

**Affiliations:** ^1^ Laboratory of Applied Mycology and Phenomics, Department of Plants and Crops, Faculty of Bioscience Engineering, Ghent University, Ghent, Belgium; ^2^ Plant Sciences Unit, Flanders Research Institute for Agriculture, Fisheries and Food (ILVO), Merelbeke, Belgium; ^3^ Laboratory of Environmental Biotechnology, Department of Applied Biosciences, Faculty of Bioscience Engineering, Ghent University, Ghent, Belgium

**Keywords:** biocontrol, streptomyces, *Fusarium graminearum*, Fusarium head blight (FHB), wheat, spicosphere microbiome, amplicon sequencing, correlation network analysis

## Abstract

**Introduction:**

Fusarium Head Blight (FHB) is a major fungal disease in wheat caused by *Fusarium graminearum*, inducing severe yield losses. Biological control agents (BCAs) can be an effective and sustainable approach to mitigate this phytopathogen. In this study, *Streptomyces rimosus* subsp. *rimosus* LMG19352 was used as a BCA to mitigate *F. graminearum* on wheat ears. Moreover, we aimed to assess the impact of BCA inoculation on non-target microorganisms present on the wheat spikes. Therefore, we evaluated shifts in the fungal and bacterial spicosphere microbiome (i) over time from flowering to mid-grain filling stage and (ii) across inoculations with *F. graminearum* and/or *S. rimosus* subsp. *rimosus* LMG19352.

**Methods:**

FHB symptoms were determined by multispectral imaging, and Illumina MiSeq was used to amplify 16S V3-V4 rDNA for bacteria and ITS2 for fungi, whereafter a correlation network analysis was performed.

**Results:**

The biocontrol potential of *S. rimosus* subsp. *rimosus* LMG19352 against *F. graminearum* was confirmed, as FHB symptoms were significantly reduced. Based on the microbial abundances, *S. rimosus* subsp. *rimosus* LMG19352 compensated for shifts in the spicosphere microbiome community induced by FHB. These results were supported by a network analysis, revealing a more complex and stable microbiome in the presence of the BCA compared to the infected control.

**Discussion:**

To our knowledge, this study is the first to reveal the potential of a bacterial BCA to temper shifts in the wheat microbiome caused by a phytopathogen, and thereby acting as a promising BCA.

## 1 Introduction

Wheat (*Triticum aestivum* L.) is an essential crop in global food production and is ranked as the second most widely grown cereal in the world in terms of production quantity (Food and Agriculture Organization of the United Nations, 2022). Fusarium Head Blight (FHB), caused by a complex of up to 17 *Fusarium* species within the FHB species complex, is one of the most economically important fungal diseases in wheat worldwide (Karlsson et al., 2021). FHB causes severe yield losses, and results in reduced grain quality and seed germination. Additionally, toxigenic *Fusarium* spp. can produce harmful mycotoxins as secondary metabolites that pose health risks to animals and humans when contaminated grain batches are consumed. The predominant causal agent of FHB worldwide is considered to be *Fusarium graminearum* which is listed as one of the top 10 fungal plant pathogens in the world (Dean et al., 2012).

To secure food and feed availability and safety, mitigation strategies to control *F. graminearum* are needed. Diverse good agricultural practices and the use of resistant cultivars have been proven to reduce the incidence of FHB (Bai et al., 2018; Shah et al., 2018). Though, in recent decades, crop protection strategies against FHB are heavily dependent on the utilization of synthetic fungicides (Pirgozliev et al., 2003). However, there are both scientific and public concerns about the environmental impact of these chemical fungicides and the potential development of pathogen resistance (Lucas et al., 2015). Additionally, their usage is limited within Europe due to strict regulations that were set by the European Green Deal (European Commission, 2020). Therefore, there is an urgent need for sustainable alternatives such as biological control agents (BCAs) to control pathogens like *F. graminearum* and hence secure grain yield and quality. Actinobacteria, and more specifically *Streptomyces* spp., are regularly reported as BCAs and have been described to exhibit a wide variety of potential biocontrol activities such as mycoparasitism, production of antibiotics and production of cell wall degrading enzymes (Palaniyandi et al., 2013; Risoli et al., 2024).

Traditionally, scientific studies on plant diseases have predominantly focused on single pathogenic strains whereas in natural environments, microbes in ecological sites are always part of an interacting microbial community (Lamichhane and Venturi, 2015). In an agricultural context, this complexity is further increased when a BCA is added to suppress or control the pathogen. These BCAs often employ modes of action such as direct antagonism or induced resistance to reduce the pathogen, yet the impact of these interventions on the endogenous microbiome is poorly understood. The advent of next-generation sequencing has resulted in a rise towards studies considering the entire microbiome, aiming to map these complex multipartite interactions (Massart et al., 2015). On the one hand, the endogenous plant microbiome can engage in several beneficial effects on plant health including increased nutrient acquisition, plant development and defense responses (Chialva et al., 2022; Müller et al., 2016; Trivedi et al., 2020), although interactions with the host plant can also be neutral or even detrimental (Xu et al., 2022). On the other hand, several studies have shown that plant-microbe interactions are bidirectional, indicating that plants can also attract beneficial microorganisms and exclude potential pathogens and thereby change the microbial community in response to biotic stress (Santoyo, 2022; Schulz-Bohm et al., 2018). Consequently, the host plant and its associated microbiome are often considered as a single functional entity, referred to as the holobiont (Hassani et al., 2018; Vandenkoornhuyse et al., 2015). To disentangle the intricate relationships in microbial communities, correlation network analyses are valuable tools for identifying co-occurrence patterns and interactions between microbial taxa under different conditions (Barberán et al., 2012; Berry and Widder, 2014; Widder et al., 2016). Integrating a network-based methodology to evaluate the effect of a third-party organism, i.e. a BCA, enhances our understanding of the holobiont and pinpoints critical microbial interactions and shifts.

Most research regarding the holobiont has focused on the rhizosphere microbiome, while comparatively less is known about the characteristics and ecological functions of microorganisms inhabiting the phyllosphere – the aerial, above-ground plant parts such as leaves, stems, flowers and fruits. Nonetheless, the phyllosphere microbiome has recently been shown to affect the food security and productivity of agricultural crops by biologically controlling phytopathogens. More specifically, Kavamura et al. (2021) introduced the term “spicosphere” as a part of the phyllosphere to describe the niche around wheat spikes or ears, which provides an important habitat for pathogenic and beneficial microorganisms living in and on the surfaces of the rachis and spikelets (Kavamura et al., 2021). However, despite the fact that spikes are the primary site for FHB infection, research on the spicosphere microbiome of wheat in relation to FHB presence is limited (Alukumbura et al., 2022; Rojas et al., 2020). Firstly, Rojas et al. (2020) investigated the fungal spicosphere microbiome of healthy versus FHB-infected wheat ears via amplicon sequencing and network analyses, and concluded that pathogenic *Fusarium* spp. appear to exclude other fungi, reducing the α-diversity of infected ears. Secondly, Alukumbura et al. (2022) studied the durum wheat ear microbiome through amplicon sequencing and network analyses when *Trichoderma gamsii* T6085 was applied on the spikes as a BCA to manage FHB and observed a minimal impact on both the bacterial and fungal microbiome of the spicosphere, which may be due to treatment T6085 not reducing the *F. graminearum* abundance, although FHB severity and incidence were significantly reduced.

This study aimed to investigate the impact of *Streptomyces rimosus* subsp. *rimosus* LMG19352, a direct antagonist of *F. graminearum* (Tan et al., 2021), not only in interaction with the pathogen itself and the host plant, but also in interaction with the endogenous microbiome of wheat ears under greenhouse conditions. Understanding the impact of a direct antagonist on non-target micro-organisms living in the niche in which it is active is critical and contributes to environmental risk assessments when developing BCA formulations, as it is desirable that artificial inoculations with a BCA do not cause major shifts in the endogenous spicosphere microbiome towards other pathogens or outcompete beneficial microorganisms. In this study, a co-inoculation assay was performed in which wheat ears were inoculated with *S. rimosus* subsp. *rimosus* LMG19352 and a GFP-transformed *F. graminearum*. After 0, 7 and 14 days post infection (dpi), FHB symptom development was assessed via multispectral imaging through measurements of the plant health (photosynthetic efficiency in photosystem II, F_v_/F_m_) and *F. graminearum* growth (cGFP). This *in planta* experiment was complemented with amplicon sequencing and correlation network analyses to investigate the impact on the fungal and bacterial community structure and composition with the aim to (i) explore changes in the spicosphere microbiome over time from flowering to mid-grain filling stage, and (ii) evaluate shifts in the spicoshpere microbiome following inoculation with *F. graminearum*, *S. rimosus* subsp. *rimosus* LMG19352, or co-inoculation with both.

## 2 Materials and methods

### 2.1 Fungal strain and conidia spore suspension

An in house GFP transformant of *Fusarium graminearum* PH-1 (Tan et al., 2020) was grown on potato dextrose agar (PDA, Sigma-Aldrich) for 14 days at 21°C under a regime of 12 h of dark and 12 h of combined UV-C and UV-A light (2× TUV 8W T5 and 1× TL 8W BLB; Philips). Conidia were harvested by adding a sterile solution of phosphate buffered saline (PBS) with 0,01% (v/v) Tween 80 to the PDA plates and scrubbing the mycelium and spores with a sterile Drigalski spatula. Subsequently, the suspension was filtered over two layers of sterile Miracloth to remove the mycelium and separate the conidia. Finally, the conidiospores were counted with a haemocytometer and the spore suspension was diluted in PBS until a concentration of 1 x 10^6^ conidia/mL was reached.

### 2.2 Actinobacterial strain

*Streptomyces rimosus* subsp. *rimosus* LMG19352 was derived from the Belgian Coordinated Collections of Microorganisms (BCCM) (Ghent, Belgium). The strain was grown from its -80°C glycerol stock on tryptic soy agar (TSA) plates for 3 days at 28°C, after which a single colony was picked with a sterile inoculation loop and was grown in 5 mL tryptic soy broth (TSB) for 3 days at 28°C and 180 rpm. The bacterial suspension was then centrifuged at 4,000 rpm for 5 minutes and washed twice with PBS. Eventually, the optical density at 600 nm (OD_600_) was measured with a spectrophotometer and set to a value of ±0.6.

### 2.3 Whole plant ear assay

**Figure 1:**
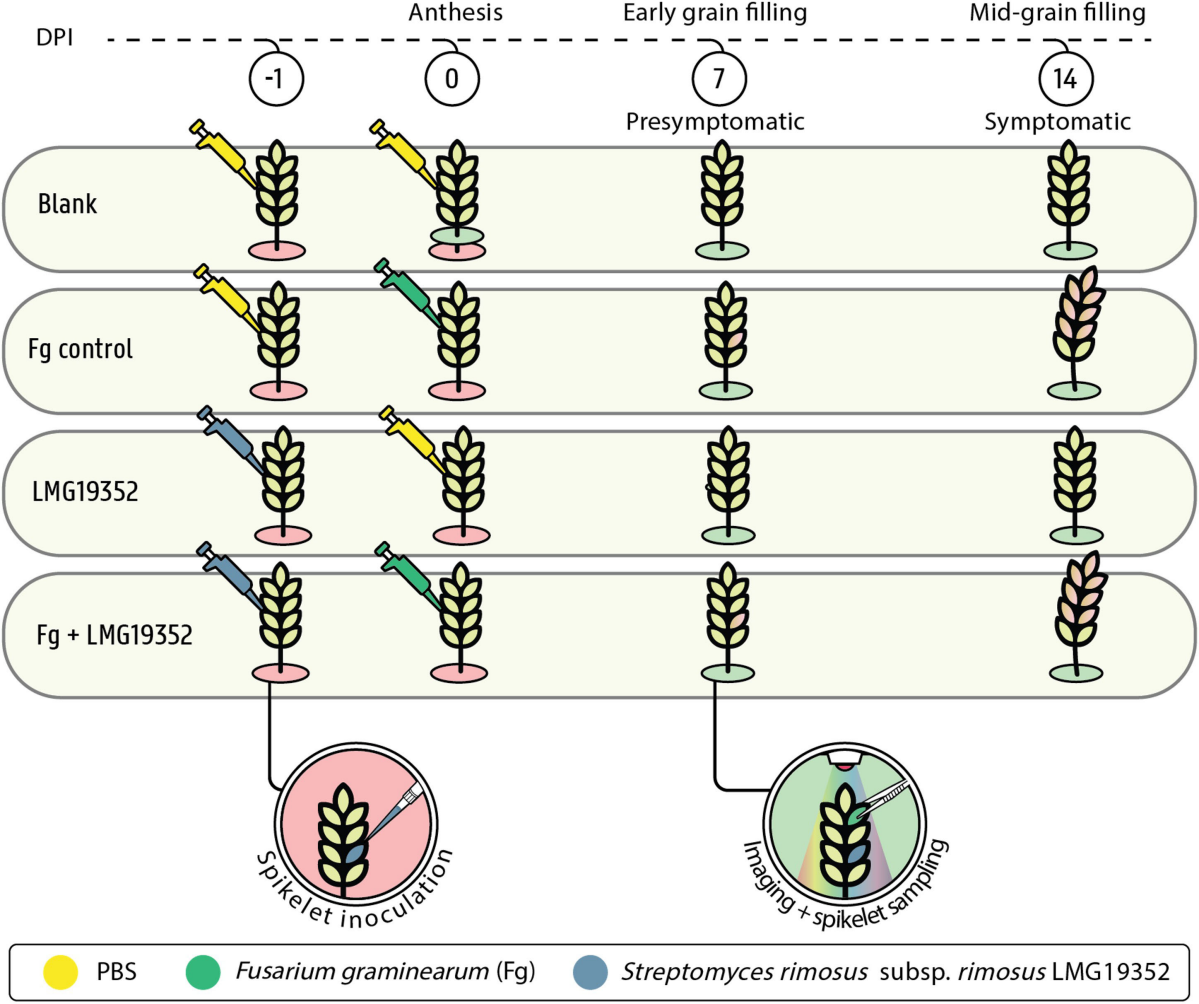
Experimental design of whole plant ear assay including PBS-inoculated wheat ears (blank), wheat ears inoculated with PBS and *F. graminearum* (Fg control), wheat ears inoculated with *S. rimosus* subsp. *rimosus* LMG19352 and PBS (LMG19352), and wheat ears co-inoculated with *S. rimosus* subsp. *rimosus* LMG19352 and *F. graminearum* (Fg+LMG19352) at one day prior (-1) and at 0 days post infection (DPI), respectively. Blank samples were taken at anthesis (0 DPI), early grain filling stage (7 DPI) and mid-grain filling stage (14 DPI), while the other treatments were only sampled at 7 and 14 DPI. Wheat ears were imaged and one spikelet, located two spikelets above the infected spikelet, was detached for sampling (i.e. sampled spikelet). Based on the imaging data, *Fusarium*-inoculated treatments were classified as presymptomatic at 7 DPI and symptomatic at 14 DPI.

Spring wheat (*Triticum aestivum* L.) cultivar Tybalt was grown under greenhouse conditions in 3-liter pots (15 cm diameter x 30 cm height) until anthesis (Zadoks Growth Scale (GS) 65). Twelve seeds were grown per pot in commercial potting soil (DCM Huis & Tuin) and mineral fertilizer was added to ensure optimal development. One spikelet per ear was used for co-inoculation with bacterial and fungal suspensions. Spikelets were inoculated with 10 µL of bacterial suspension through point inoculation 24 h before fungal inoculation (-1 dpi). One day after bacterial inoculation, the same spikelet was inoculated with 10 µL of *F. graminearum* spore suspension (0 dpi). An infected control, in which sterile PBS was used instead of bacterial suspensions, and a blank control, which was co-inoculated with sterile PBS, were included (**Figure 1**). After inoculation, plants were put in a custom-built growth chamber at 21°C with a photoperiod of 16 h and kept at raised relative humidity for 3 days by spraying the plants and the walls of the growth chamber with water until run-off twice a day (approximately 100% relative humidity). Samples were taken at 0, 7 and 14 dpi by detaching the ears, visualizing them through multispectral imaging, and subsequently flash freeze them in liquid nitrogen. Samples were stored at -80°C for further analysis. All treatments and controls were included in 15 randomly distributed replicates.

### 2.4 Disease progression through multispectral imaging

Disease assessment was performed as described by Tan et al. (2020). In short, a custom built multispectral imaging platform was used (CropReporter, Phenovation B.V., Wageningen, The Netherlands) to visualize diverse physiological traits in real time, based on specific absorption, reflection and emission patterns at a resolution of 6 μm. A 6Mp-16 bit camera, including a filter wheel equipped with 12 optical interference filters, allows pixel-to-pixel capturing of RGB values, a diverse set of spectral indices, chlorophyll fluorescence (F_v_/F_m_), and GFP fluorescence. The effect of disease was assessed via the photosynthetic efficiency in the electron transport chain of photosystem II in a dark adapted state (F_v_/F_m_) (Baker, 2008). Additionally, GFP tagged *F. graminearum* PH-1 was visualized and quantified by measuring GFP fluorescence, enabling the assessment of fungal growth *in planta*. The GFP signal was corrected for autofluorescence of senescing ears, resulting in the corrected GFP value (cGFP).

### 2.5 DNA extraction

Based on the results of our multispectral imaging platform, the ten most representative wheat ear samples of each treatment were selected and randomly pooled per two. Per ear, the spikelet located two spikelets above the inoculated spikelet was selected (**Figure 1**) and was ground with liquid nitrogen. A total DNA extraction was carried out using the DNeasy Plant Mini Kit (QIAGEN, The Netherlands) according to the manufacturer’s instructions with some slight adaptations. After washing the column twice with wash buffer AW2, the spin column was transferred to a new microcentrifuge tube and airdried for 1 min to make sure all the ethanol was evaporated. The volume of the elution buffer was adapted to 50 µL, and columns with elution buffer were incubated for 5 min at room temperature. Afterwards, samples were centrifuged for 1 min at 6,000 g and final steps were repeated once with the eluate. Concentration of extracted DNA was evaluated using the QuantiFluor^®^ dsDNA System and the Quantus™ Fluorometer from Promega (Madison, Wisconsin, USA). The DNA extracts were finally diluted with nuclease-free water to a concentration of 5 ng/μL, unless the concentration was lower than 10 ng/µL, then no further dilution was performed.

### 2.6 Library preparation and amplicon sequencing

The bacterial V3-V4 region of the 16S rDNA was amplified by an amplicon PCR using the primer pair S-D-Bact-0341-b-S-17/S-D-Bact-0785-a-A-21 (Klindworth et al., 2013) (**Table 1**), extended with Illumina specific adaptors. The fungal ITS2 region was amplified by an amplicon PCR using the forward primer fITS7bis (adapted from Ihrmark et al. (2012)) and reverse primer ITS4NGSr (adapted from Tedersoo et al. (2014)) (**Table 1**), both extended with Illumina specific adaptors. Afterwards, an index PCR was performed to attach dual indices and Illumina sequencing adaptors to both the V3-V4 and the ITS2 fragments using the Nextera XT Index Kit (Illumina, San Diego, CA, USA). Identical PCR conditions for both PCRs were used as described by Debode et al. (2016) and PCR mastermixes for the amplicon and index PCR were prepared using KAPA HiFi HotStart ReadyMix (Kapa Biosystems, Wilmington, MA, USA) according to the manufacturer’s instructions to a total amount of, respectively, 25 µL and 50 μL per sample. Each PCR step was followed by a PCR clean-up using CleanNGS beads (CleanNA, Waddinxveen, The Netherlands). The cleaned PCR products were each time controlled for their quality via gel electrophoresis and final concentrations were measured using the QuantiFluor^®^ dsDNA System and the Quantus™ Fluorometer (Promega, Madison, Wisconsin, USA). Eventually, the barcoded libraries were diluted to 10 nM, equally pooled and sequenced on an Illumina MiSeq system (2 × 300 bp) by Admera (South Plainfield, NJ, USA).

**Table 1:** Sequences of the forward and reverse V3-V4 and ITS2 primers used to amplify the bacterial 16S rDNA and fungal ITS gene, respectively.

Region	Primer	Direction	Sequence
V3-V4	S-D-Bact-0341-b-S-17	Forward	5′-CCTACGGGNGGCWGCAG-3′
	S-D-Bact-0785-a-A-21	Reverse	5′-GACTACHVGGGTATCTAATCC-3’
ITS2	fITS7bis	Forward	5’-GTGAATCATCRAATYTTTG-3’
	ITS4NGSr	Reverse	5’-CAWCGATGAAGAACGYAG-3’

To avoid amplification of mitochondrial and plastid 16S rDNA derived from the host plant during PCR, peptide nucleic acid (PNA) clamps were added to the PCR mastermix used for amplification of V3-V4 16S rDNA (PNABio, California, USA) (**Table 2**). PNA is a synthetic DNA mimic in which the DNA deoxyribose phosphodiester backbone is replaced with a pseudopeptide backbone (Nielsen and Egholm, 1999). These PNA clamps selectively bind to a targeted region of the plant genome and inhibit amplification of this host DNA during PCR, since the PNA/DNA duplexes are thermally more stable than the DNA/DNA duplexes formed during primer annealing (Lundberg et al., 2013; Ørum et al., 1993).

**Table 2:** Sequences of peptide nucleic acid (PNA) clamps used to block the amplification of plastid and mitochondrial rDNA.

rDNA blocker	Sequence of PNA clamps
Chloroplast	5’-GGCTCAACCCTGGACAG-3’
Mitochondria	5’-GGCAAGTGTTCTTCGGA-3’

### 2.7 Sequence processing and downstream data analysis

Demultiplexing of the amplicon dataset was done by the sequencing provider. Cutadapt was used to look for primers and only keep those fragments where both primers were found at the beginning and at the end. Afterwards, primers and adapters were removed. Additionally, fragments were filtered by allowing maximum three errors, and fragments with N’s were excluded. Raw Illumina forward and reverse reads were merged using the program PEAR v.0.9.8 (Zhang et al., 2014). Dereplication, sorting, amplicon sequence variant (ASV) calling, chimera removal, and taxonomic assignment were done making use of the DADA2 algorithm v1.16 (Callahan et al., 2016). Briefly, all ambiguous bases were removed and the number of maximum expected errors for forward and reverse reads was set at three. After dereplication, reads were merged and an ASV table was built. The DADA2 AssignTaxonomy function was used to assign taxonomy to the resulting ASVs, with the SILVA database v138.1 (Quast et al., 2012) serving as a reference for V3-V4 16S rRNA gene sequences and the UNITE database v9.0 for fungal ASVs. The resulting count table was further filtered based on length and taxonomy. A length cut-off for the merged sequences for V3-V4 and ITS2 were set between 400-450 bp and 235-450 bp, respectively. Taxa unassigned to the kingdom of Bacteria, unidentified on phylum level, assigned to the class Chloroplast or family Mitochondria were excluded from the bacterial dataset. Taxa unassigned to the kingdom of Fungi and/or unidentified on class level were excluded from the fungal dataset. The filtered count tables were used for statistical analyses.

### 2.8 Statistical analyses

#### 2.8.1 Downstream processing of the ASV table

Statistical analyses on the ASV table were done in R and RStudio version 4.3.1 (Posit Team, 2023; R Core Team, 2023) at a significance level of α = 0.05.

Chao1 species richness, Shannon diversity index, and Pielou’s evenness were determined for fungi and bacteria per treatment and per timepoint. Assumptions regarding normality and homoscedasticity were verified by a Shapiro-Wilk and Levene test, respectively, after which a one-way ANOVA followed by a Tukey or Dunnett T3 *post hoc* test was used to examine significant differences across treatments. Plant health parameters (F_v_/F_m_ and cGFP) were correlated to these three α-diversity measures by means of Spearman correlations using the packages *Hmisc* and *corrplot* (Harrell, 2024; Wei and Simko, 2021).

Similarity and clustering relationships of distinctive ASVs for each timepoint or treatment were visualized in a Venn diagram using the *ggVennDiagram* package (Gao and Dusa, 2024). Taxa were analyzed at class level using the *phyloseq* package (McMurdie and Holmes, 2013). Bacterial and fungal abundances were filtered by only keeping those classes which were present with a minimal relative abundance of 0.05% in at least five samples. Statistical analysis on the relative abundance table was done through a Kruskal-Wallis test followed by a Dunn *post hoc* test with Bonferroni correction.

To reduce the dimensionality of the dataset, a Principal Component Analysis (PCA) was performed using the *FactoMineR* package in R (Husson et al., 2024) and the first five principal components were retained. Afterwards, a Hierarchical clustering on Principal Components (HCPC) was performed through the function HCPC. Briefly, the HCPC function uses the Ward’s criterion (minimal within-cluster variance) for hierarchical clustering. The suggested number of clusters was not pre-specified, allowing the algorithm to automatically determine the optimal number of clusters based on the dataset.

#### 2.8.2 Correlation network analysis

Spearman’s rank-order correlation coefficients were calculated between the community relative abundance matrices using the rcorr-function within R package *Hmisc* (Harrell, 2024). Bootstrap resampling (n = 1000) was performed to estimate the stability and variability of the correlations. The bootstrap bias and confidence interval statistics were used to filter the original Spearman’s correlation coefficient matrix, in which (i) the confidence interval around the bootstrap value should not include zero, and (ii) the bootstrap bias values should not exceed the standard error. Correlation networks were constructed using the R package *network* (Butts, 2008) and then visualized using the ggnet-function. Centrality measures (degree, betweenness) and most other topological parameters (nodes, edges, density, clustering, shortest path length) were determined using the R package *sna* (Butts, 2023) while assortativity and modularity were calculated using the R package *igraph* (Csárdi et al., 2024).

#### 2.8.3 Image processing and data analysis

Multispectral images of wheat ears were processed using the DataAnalysis software v.5.4.4 (PhenoMate; PhenoVation, Wageningen, The Netherlands) by filtering the background based on chlorophyll values (Chl > 200) and a minimal pixel size of 1000. Data were gathered at specific regions of interest (ROI, 300 x 350 pixels) situated at the infected spikelet, and the sampled spikelet. For each ROI, the chlorophyll fluorescence (F_v_/F_m_ value) and the cGFP signal were determined. Downstream processing of the multispectral data was done using IBM SPSS statistics version 28. Assumptions regarding normality and homoscedasticity were verified by a Shapiro-Wilk and Levene test, respectively. Afterwards, a one-way ANOVA followed by a Tukey *post hoc* test was performed for multiple comparison. All tests were conducted with a significance level of α = 0.05. For plots generation, RStudio and the package *ggplot2* were used (Posit Team, 2023; Wickham, 2016).

## 3 Results

### 3.1 *Streptomyces rimosus* subsp. *rimosus* LMG19352 diminishes Fusarium Head Blight symptoms on wheat ears at the *Fusarium graminearum* inoculation site

**Figure 2:**
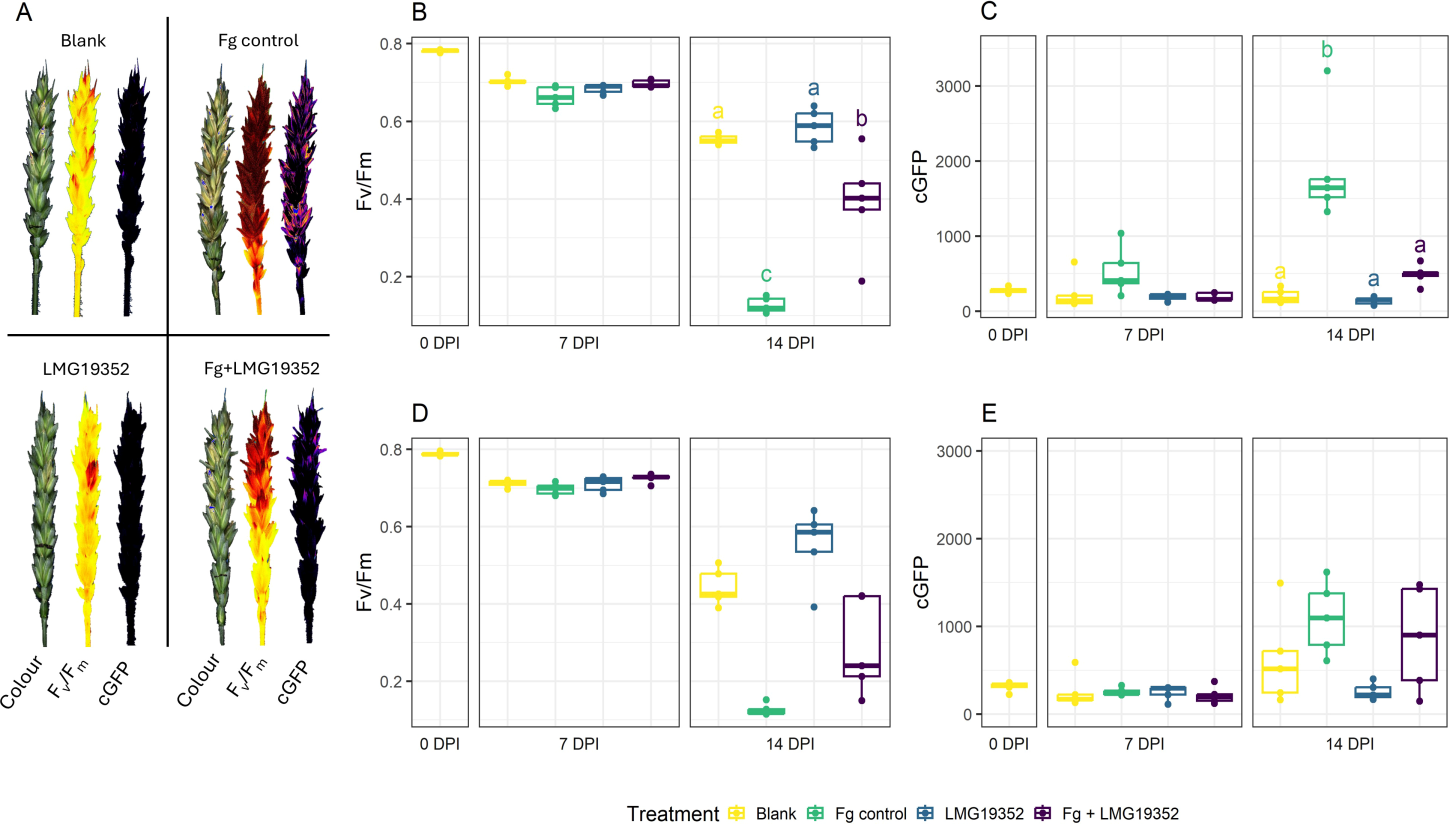
Multispectral images (colour, chlorophyll fluorescence (F_v_/F_m_), and corrected GPF-signal (cGFP)) of uninoculated wheat ears (blank), wheat ears inoculated with *F. graminearum* (Fg control), wheat ears inoculated with *S. rimosus* subsp. *rimosus* LMG19352 (LMG19352), and wheat ears co-inoculated with *F. graminearum* and *S. rimosus* subsp. *rimosus* LMG19352 (Fg + LMG19352) at 14 days post infection (DPI) **(A)**. F_v_/F_m_ **(B, D)** and cGFP **(C, E)** of uninoculated wheat ears (blank), wheat ears inoculated with *F. graminearum* (Fg control), wheat ears inoculated with *S. rimosus* subsp. *rimosus* LMG19352 (LMG19352), and wheat ears co-inoculated with *F. graminearum* and *S. rimosus* subsp. *rimosus* LMG19352 (Fg + LMG19352) evaluated at the infection spikelet **(B, C)** and sampled spikelet **(D, E)** at 0, 7 and 14 DPI; n = 5 biological replicates. Significant differences are indicated per timepoint by means of significance letters as determined through a one-way ANOVA and Tukey *post hoc* test at a 95% confidence level. When no significant differences were observed between treatments at a certain timepoint, significance letters were omitted.

The impact of *S. rimosus* subsp. *rimosus* LMG19352 on a *F. graminearum* infection was investigated on wheat ears. After 0, 7 and 14 dpi, FHB symptom development was measured via F_v_/F_m_ and cGFP assessing plant health and active fungal biomass, respectively (**Figure 2**). At 7 dpi, the fungal pathogen was still in its presymptomatic stage, as no significant differences across treatments were observed, while at 14 dpi clear symptom development was established.

At 14 dpi, the infected spikelet of wheat ears co-inoculated with BCA *S. rimosus* subsp. *rimosus* LMG19352 and *F. graminearum* resulted in a significant decrease in FHB symptom development, hallmarked by significantly higher F_v_/F_m_ values as compared to the infected *F. graminearum* control (**Figure 2B**; p < 0.001). Additionally, a significant decrease in cGFP value was detected, indicating a lower biomass accumulation of *F. graminearum* at that timepoint (**Figure 2C**; p < 0.001). These observations both confirmed the biocontrol activity of strain LMG19352 as earlier observed by Tan et al. (2021). Additionally, upon comparison between the co-inoculated ears with the uninfected, LMG19352-treated control at 14 dpi, no significant differences were observed between the cGFP values, suggesting a complete loss of actively growing *F. graminearum* on the infected spikelet after 14 dpi (p = 0.995). Eventually, no phytotoxic effects were observed in wheat ears inoculated with strain LMG19352 alone.

Assessment of symptom development of the sampled spikelets, located two spikelets above the infection site (**Figure 1**), resulted in a similar trend as observed for the infected spikelets, but did not result in any significant differences (**Figures 2D, E**). At 14 dpi, the co-inoculated treatment showed a higher variation and a lower median of F_v_/F_m_ values in the sampled spikelets (**Figure 2D**) when compared to the results of the inoculated spikelets (**Figure 2B**). In addition, the opposite effect was observed for the median cGFP value (**Figures 2C, E**). These results both suggest that the biocontrol effect of strain *S. rimosus* subsp. *rimosus* LMG19352 diminishes when moving further away from the inoculation site.

### 3.2 Fungal alpha diversity of the spicosphere microbiome is reduced upon Fusarium Head Blight symptom development

The fungal and bacterial community structures on wheat ears inoculated with *S. rimosus* subsp. *rimosus* LMG19352 and/or *F. graminearum* were examined at 0, 7 and 14 dpi. In the original, unfiltered ASV tables, a total of 10,911,934 and 2,506,704 reads were detected and clustered into 6,481 and 6,063 ASVs for the fungal and bacterial dataset, respectively. After filtering the dataset based on ASV length and taxonomy, the fungal and bacterial datasets contained 1,445 and 759 ASVs, respectively. Chao1 richness, Pielou’s evenness and the Shannon diversity index were calculated for both filtered datasets (**Table 3**). No significant differences in α-diversity measures across treatments were observed in the bacterial dataset. However, at 7 dpi, the mean chao1 richness was higher for treatment LMG19352 compared to the other treatments, due to an extreme value of 217 species counts in one of the replicates, although not significant. In contrast, analysis of the fungal dataset at 7 dpi showed that richness, evenness and diversity were significantly lower in treatment LMG19352 when compared to the blank control (p = 0.034, 0.021 and 0.01, respectively). Both fungal and bacterial differences in α-diversity were restored at 14 dpi. The *F. graminearum* infected control at 14 dpi showed a significantly lower richness, evenness and diversity in the fungal dataset compared to the blank (p = 0.008, 0.011 and 0.01, respectively). This trend was not yet observed at the presymptomatic timepoint (i.e. 7 dpi), indicating that the loss in diversity is associated with symptom development of *F. graminearum*. This assumption was supported by a significant positive correlation of the fungal richness, evenness, and diversity with the plant health status (F_v_/F_m_) in both infected and sampled spikelets. Additionally, fungal richness, evenness and diversity were negatively correlated with *F. graminearum* growth (cGFP) in both infected and sampled spikelets, however the correlation between fungal richness and cGFP was not significant (**Supplementary Figure 1**), indicating that lower fungal diversity is associated with increased biomass of the inoculated *F. graminearum*. Eventually, co-inoculated samples displayed intermediate α-diversity values between the blank and infected control at 14 dpi, indicating a partial recovery of α-diversity towards the original state, similar to the trend observed for FHB symptoms.

**Table 3:** Chao1 richness, Pielou’s evenness index and Shannon’s diversity index of fungi and bacteria at 0, 7 and 14 days post infection (DPI) on uninoculated wheat ears (blank), wheat ears inoculated with *F. graminearum* (Fg control), wheat ears inoculated with *S. rimosus* subsp. *rimosus* LMG19352 (LMG19352), and wheat ears co-inoculated with *F. graminearum* and *S. rimosus* subsp. *rimosus* LMG19352 (Fg+LMG19352).

		Fungi			Bacteria		
DPI	Treatment	Chao1 richness	Pielou’s evenness	Shannon diversity	Chao1 richness	Pielou’s evenness	Shannon diversity
0	Blank	150 ± 94	0.61 ± 0.08	2.99 ± 0.72	21 ± 23	0.82 ± 0.03	2.22 ± 0.73
7	Blank	124 ± 49^a^	0.52 ± 0.07^a^	2.49 ± 0.49^a^	13 ± 5	0.68 ± 0.33	1.70 ± 0.82
	Fg control	107 ± 16^a,b^	0.47 ± 0.12^a,b^	2.21 ± 0.56^a,b^	24 ± 8	0.83 ± 0.12	2.60 ± 0.40
	LMG19352	71 ± 11^b^	0.33 ± 0.12^b^	1.42 ± 0.53^b^	60 ± 88	0.57 ± 0.24	1.89 ± 0.76
	Fg+LMG19352	100 ± 14^a,b^	0.54 ± 0.03^a^	2.46 ± 0.13^a^	20 ± 14	0.90 ± 0.03	2.59 ± 0.58
14	Blank	95 ± 2^a^	0.46 ± 0.13^a^	2.09 ± 0.59^a^	18 ± 10	0.72 ± 0.25	1.93 ± 0.62
	Fg control	64 ± 8^b^	0.09 ± 0.03^b^	0.37 ± 0.13^b^	27 ± 6	0.85 ± 0.08	2.76 ± 0.17
	LMG19352	103 ± 11^a^	0.42 ± 0.18^a,b^	1.94 ± 0.83^a,b^	22 ± 5	0.54 ± 0.29	1.66 ± 0.93
	Fg+LMG19352	86 ± 22^a,b^	0.34 ± 0.23^a,b^	1.53 ± 1.06^a,b^	20 ± 4	0.83 ± 0.10	2.46 ± 0.34

Values represent means ± standard deviation of 5 biological replicates. Significant differences are indicated per timepoint by means of significance letters as per one-way ANOVA and Tukey or Dunnett T3 *post hoc* test at a 95% confidence level.

### 3.3 Shifts in community composition of the spicosphere microbiome

The fungal and bacterial community compositions of the spicosphere microbiome were investigated over time (0, 7 and 14 dpi), and across treatments on uninoculated wheat ears (blank), wheat ears inoculated with *F. graminearum* (Fg control), wheat ears inoculated with *S. rimosus* subsp. *rimosus* LMG19352 (LMG19352) and wheat ears co-inoculated with *F. graminearum* and *S. rimosus* subsp. *rimosus* LMG19352 (Fg + LMG19352).

Before filtering, the fungal abundance dataset contained 7 phyla, 33 classes, 82 orders, 201 families and 341 genera, while the bacterial abundance dataset contained 24 phyla, 46 classes, 107 orders, 152 families and 219 genera. The dataset was filtered to only retain the relevant phyla, classes, orders, families or genera with a minimal abundance of 0.05% in at least 5 samples, after which the fungal and bacterial dataset respectively consisted of 3 and 10 phyla, 11 and 15 classes, 25 and 24 orders, 32 and 34 families, 31 and 24 genera and 133 and 26 ASVs.

#### 3.3.1 Shifts in community composition over time

**Figure 3:**
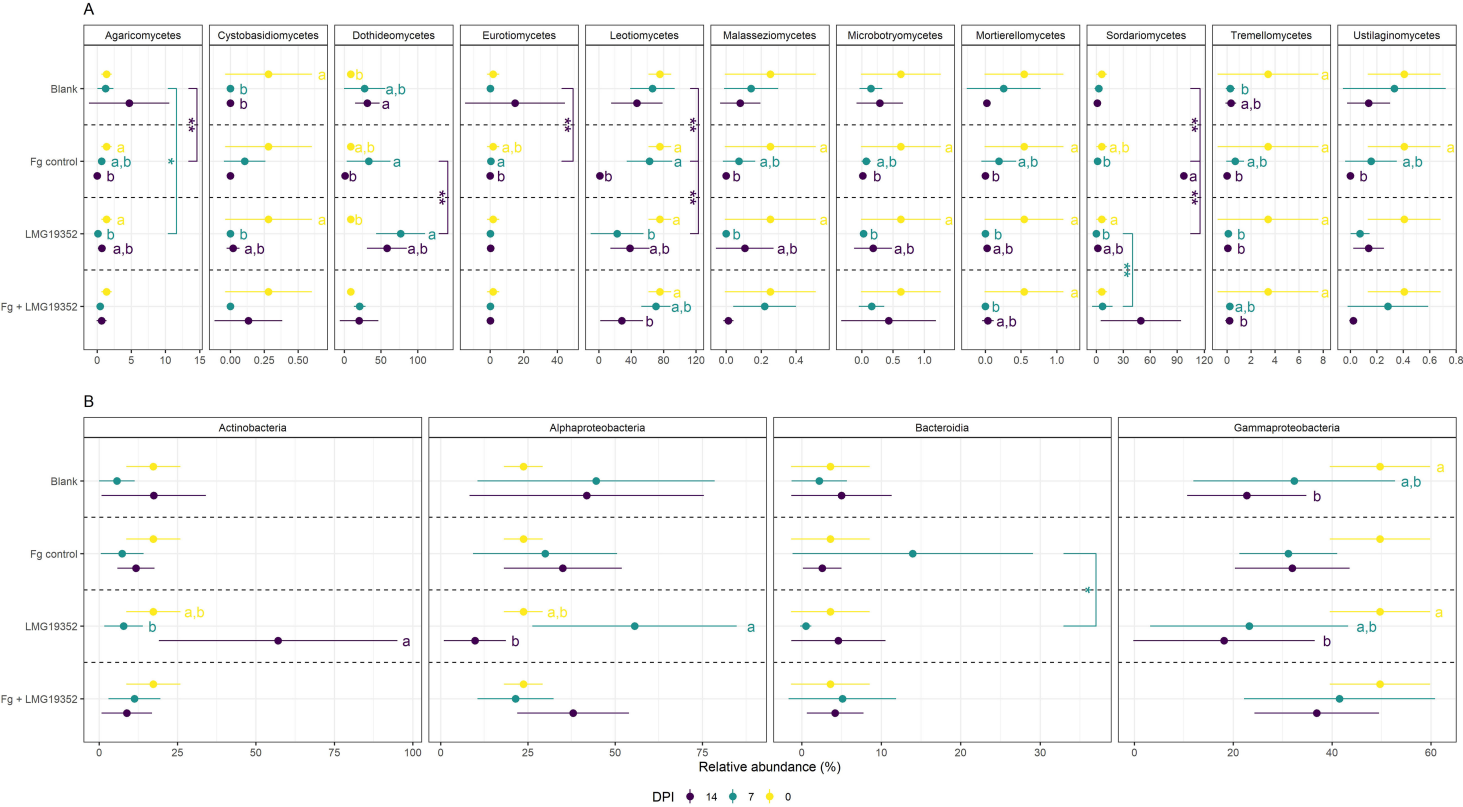
Relative abundances (%) of fungal **(A)** and bacterial **(B)** classes on uninoculated wheat ears (blank), wheat ears inoculated with *F*. *graminearum* (Fg control), wheat ears inoculated with *S. rimosus* subsp. *rimosus* LMG19352 (LMG19352) and wheat ears co-inoculated with *F*. *graminearum* and *S. rimosus* subsp. *rimosus* LMG19352 (Fg + LMG19352). Samples were collected at 0, 7 and 14 days post infection (DPI). Only classes with at least one significant difference between timepoints or treatments are shown. Values represent means ± standard deviation of 5 replicates. Significant differences between timepoints are indicated by significance letters at a 95% confidence level. Significant differences between treatments are indicated by asterisks: *p < 0.05; **p < 0.025. When no significant differences were observed across treatments or between timepoints, significance markers were omitted.

In the unfiltered dataset of fungal and bacterial ASVs together, most ASVs were specific for a certain timepoint and only 176 ASVs were shared between all three timepoints, indicating shifts in the spicosphere community composition over time (**Supplementary Figure 2A**). To investigate how the community composition of the spicosphere microbiome changes over time, temporal shifts in relative abundance at the taxonomical level of class in the filtered dataset were determined for each treatment (**Figure 3**; **Supplementary Figure 3**).

##### 3.3.1.1 Changes in the endogenous spicosphere microbiome from flowering to mid-grain filling stage are minimal

To investigate temporal changes in the endogenous microbiome on wheat ears, the differences in community composition of the blank control were analyzed across three timepoints from flowering (i.e. 0 dpi) over early grain filling stage (i.e. 7 dpi) to mid-grain filling stage (i.e. 14 dpi) (**Figure 3**). Only a few significant differences in relative abundance were observed, which might be due to the variability within the samples. Significant fungal differences were observed for Dothideomycetes between 0 and 14 dpi and for Tremellomycetes between 0 and 7 dpi. Additionally, a significant decrease in Cystobasidiomycetes was found between 0 and 7 dpi on the one hand and 0 and 14 dpi on the other hand, as this fungal class was no longer found in samples taken at the latest two timepoints. For the bacterial abundance dataset, a single significant difference was detected for Gammaproteobacteria between 0 and 14 dpi. These results indicate that the endogenous spicosphere microbiome changes over time. Though, the overall microbiome composition appears to remain relatively stable, since only a few significant changes could be observed at class level in the blank control samples.

##### 3.3.1.2 The fungal spicosphere microbiome upon *Fusarium graminearum* colonization changes in parallel with Fusarium Head Blight symptom development, but the bacterial microbiome remains stable

Compositional changes within wheat ears inoculated with *F. graminearum* (Fg control) over time were investigated to check how the fungal and bacterial microbiome of wheat ears changes from a non-inoculated stage (i.e. 0 dpi) over a presymptomatic stage (i.e. 7 dpi) to a symptomatic stage where FHB symptoms were clearly visible (i.e. 14 dpi) (**Figure 3**). The abundance of Agaricomycetes, Malasseziomycetes, Microbotryomycetes, Mortierellomycetes, Tremellomycetes and Ustilaginomycetes significantly declined from 0 to 14 dpi, with intermediate levels observed at 7 dpi. Similar results were observed for Cystobasidiomycetes, although differences in relative abundance between timepoints were not significant. Oppositely, Leotiomycetes became less and less abundant over time, though their abundance at 0 and 7 dpi was significantly different from 14 dpi but not from each other. The relative abundance of Dothideomycetes and Eurotiomycetes significantly decreased between 7 and 14 dpi, whereas that of Sordariomycetes (the class to which *Fusarium* spp. belong) significantly increased. When looking at genus level, the observed increase in relative abundance of Sordariomycetes is largely driven by *Fusarium*. Contrastingly to the changes in community composition of the fungal microbiome, where significant differences in fungal abundances over time were observed for almost all fungal classes, no significant differences were detected in bacterial abundances over time.

Overall, it can be concluded that there were no significant shifts between the non-inoculated stage (i.e. 0 dpi) and presymptomatic stage (i.e. 7 dpi) in the spicosphere microbiome, but that 10 out of 11 fungal classes had significantly different abundances when comparing the non-inoculated stage (i.e. 0 dpi) versus the symptomatic stage (i.e. 14 dpi). From these results it can be suggested that not the inoculation with *F. graminearum* itself but rather the development of FHB symptoms causes the shifts in the fungal spicosphere microbiome. Additionally, nor the inoculation nor the induction of symptoms had a significant impact on the bacterial spicosphere microbiome.

##### 3.3.1.3 The spicosphere microbiome changes over time upon inoculation with biocontrol agent *Streptomyces rimosus* subsp. *rimosus* LMG19352

To investigate how the microbiome of wheat ears inoculated with *S. rimosus* subsp. *rimosus* LMG19352 shifts from flowering (i.e. 0 dpi) to mid-grain filling stage (i.e. 14 dpi), significant community changes over time were identified. Out of all 11 fungal classes, only two (Eurotiomycetes and Ustilaginomycetes) did not show any significant differences between timepoints. Nearly all other fungal classes were most abundant at 0 dpi, except for Dothideomycetes. Their relative abundance was lowest at 0 dpi and was significantly increased at 7 dpi followed by a decrease in abundance at 14 dpi, however not significant, to an intermediate level. Contrastingly, the relative abundance of Agaricomycetes, Cystobasidiomycetes, Leotiomycetes, Malasseziomycetes, Microbotryomycetes, Mortierellomycetes and Sordariomycetes was significantly decreased from 0 to 7 dpi and increased again at 14 dpi, however the abundance at 14 dpi was not significantly different from those at both 0 and 7 dpi. Additionally, the relative abundance of Tremellomycetes at both 7 and 14 dpi was significantly reduced when compared to their relative abundance observed at 0 dpi. Regarding the bacterial community, Actinobacteria became significantly more abundant between 7 and 14 dpi, while Alphaproteobacteria became significantly less abundant. Both bacterial classes showed intermediate relative abundances at 0 dpi, which did not differ significantly from the values observed at both 7 and 14 dpi. Eventually, the relative abundance of Gammaproteobacteria declined over time, with a significant difference between the abundances observed at 0 and 14 dpi.

Overall, it can be concluded that inoculation with *S. rimosus* subsp. *rimosus* LMG19352 caused significant shifts in the fungal spicosphere microbiome, mostly when comparing flowering stage (i.e. 0 dpi) with early grain filling stage (i.e. 7 dpi). However, most changes were partly restored again at mid-grain filling stage (i.e. 14 dpi). Furthermore, it can be concluded that inoculation with *S. rimosus* subsp. *rimosus* LMG19352 also resulted in significant changes in the bacterial spicosphere microbiome. The high abundance of Actinobacteria (the class to which *Streptomyces* spp. belong) at mid-grain filling stage (i.e. 14 dpi) might have originated from the inoculation with strain LMG19352, as the increase at genus level was mostly derived from *Streptomyces*. This suggests that the biocontrol strain may outcompete other microorganisms such as members of Alphaproteobacteria and Gammaproteobacteria, since the abundances of the latter two were reduced at that timepoint. Combining the results of fungal and bacterial shifts suggests that fungi may respond more rapidly to inoculation with *S. rimosus* subsp. *rimosus* LMG19352 as most fungal differences were observed at early grain filling stage (i.e. 7 dpi) compared to flowering (i.e. 0 dpi), whereas bacterial shifts were detected at mid-grain filling stage (i.e. 14 dpi). As fungal shifts were partially restored by mid-grain filling (i.e. 14 dpi), it is possible that bacterial shifts may also be restored at a later stage. Alternatively, this pattern might suggest that *S. rimosus* subsp. *rimosus* LMG19352 partially compensates for the early fungal shifts, as the Actinobacterial increase at 14 dpi coincided with a shift in the fungal microbiome toward its original state in non-inoculated wheat ears. However, in this assumption, the underlying causes of these early fungal shifts at 7 dpi remain unclear.

##### 3.3.1.4 The biocontrol agent *Streptomyces rimosus* subsp. *rimosus* LMG19352 tempers shifts in the spicosphere microbiome induced by Fusarium Head Blight symptoms

Temporal shifts in the fungal and bacterial spicosphere microbiome of co-inoculated wheat ears were studied to investigate the impact and biocontrol potential of *S. rimosus* subsp. *rimosus* LMG19352 in the presence of a *F. graminearum* infection during a non-inoculated (i.e. 0 dpi), presymptomatic (i.e. 7 dpi), and symptomatic stage (i.e. 14 dpi). Leotiomycetes and Tremellomycetes became less abundant over time with a significant decrease in relative abundance between 0 and 14 dpi. A significant decrease in relative abundance of Mortierellomycetes was observed between 0 and 7 dpi, whereas their value at 14 dpi was not significantly different from both 0 and 7 dpi. Additionally, Sordariomycetes became more abundant over time, although not significant. When looking at the relative abundance of bacterial classes, no significant differences over time were observed in the co-inoculated wheat ears.

Overall, co-inoculating wheat ears with *S. rimosus* subsp. *rimosus* LMG19352 and *F. graminearum* resulted in relatively few significant shifts in the spicosphere microbiome over time. These findings suggest that inoculation of wheat ears with BCA *S. rimosus* subsp. *rimosus* LMG19352 partially compensates shifts in the spicosphere microbiome caused by FHB symptoms, as the development of FHB symptoms following a single inoculation with *F. graminearum* led to numerous shifts in the fungal spicosphere microbiome (3.3.1.2).

#### 3.3.2 Shifts in community composition across treatments

In the unfiltered dataset of fungal and bacterial ASVs together, most ASVs were shared between all treatments. Additionally, almost no ASVs were shared between two or three treatments, while many treatment specific ASVs were detected, indicating changes in spicosphere community composition across treatments (**Supplementary Figure 2B**). To investigate how the spicosphere microbiome changes after an inoculation with *S. rimosus* subsp. *rimosus* LMG19352 and/or *F. graminearum*, differences in community composition between different treatments were studied in the filtered dataset at class level (**Figure 3**; **Supplementary Figure 3**).

Firstly, the impact of a *F. graminearum* inoculation on the spicosphere microbiome was assessed by comparing the blank control with the infected control (Fg control) at both 7 and 14 dpi (**Figure 3**). On the one hand, the abundance of Sordariomycetes, which includes the genus *Fusarium*, significantly increased in the infected control at 14 dpi compared to the blank control, most likely due to the inoculation with *F. graminearum*. On the other hand, Agaricomycetes, Eurotiomycetes and Leotiomycetes had similar abundances to the blank control at 7 dpi, though were significantly less abundant at 14 dpi. These findings indicate competitive interactions between Sordariomycetes and those other three fungal classes. No significant changes in relative abundance of bacterial classes were found between both treatments, indicating that *F. graminearum* only impacts the fungal spicosphere microbiome, which is in accordance with the observed shifts in community composition over time (3.3.1.2).

Secondly, potential shifts in the spicosphere microbiome induced by inoculation with *S. rimosus* subsp. *rimosus* LMG19352 were assessed and compared to the blank (**Figure 3**). At 7 dpi, wheat ears inoculated with strain LMG19352 were significantly less abundant in Agaricomycetes than the blank control samples. No additional significant differences in fungal or bacterial taxa composition were found on the taxonomic level of classes when compared to the blank control.

Thirdly, significant differences between wheat ears co-inoculated with *F. graminearum* and *S. rimosus* subsp. *rimosus* LMG19352 and the other treatments were unraveled (**Figure 3**). First, compared to treatment with LMG19352 alone, Sordariomycetes were significantly more abundant at 7 dpi in the co-inoculated ears while no significant differences were observed between the co-inoculated treatment and the infected control, or the LMG19352 treated wheat ears at 14 dpi. This shows that co-inoculation resulted in an intermediate effect on Sordariomycetes. Since changes within the class of Sordariomycetes could have largely been attributed to shifts in the genus *Fusarium*, these results imply that application of BCA *S. rimosus* subsp. *rimosus* LMG19352 reduces *F. graminearum* abundance, although not significant.

#### 3.3.3 Hierarchical clustering on principal components unravels differences in the overall community composition across treatments and timepoints

**Figure 4:**
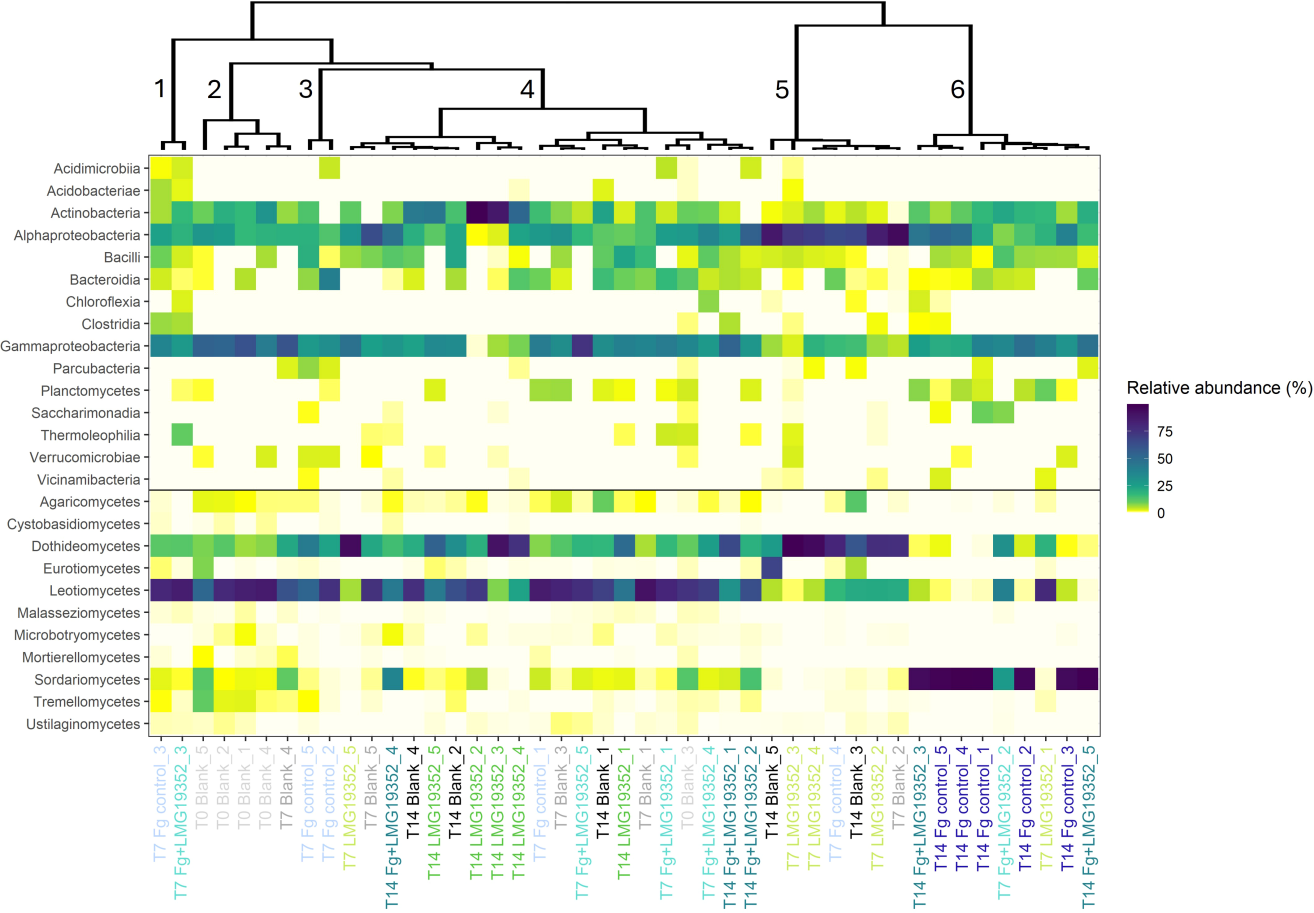
Heatmap of relative abundances of fungal and bacterial classes on uninoculated wheat ears (blank), wheat ears inoculated with *F. graminearum* (Fg control), wheat ears inoculated with *S. rimosus* subsp. *rimosus* LMG19352 (LMG19352) and wheat ears co-inoculated with *F. graminearum* and *S. rimosus* subsp. *rimosus* LMG19352 (Fg+LMG19352). Samples were collected at 0, 7 and 14 days post infection (T0, T7 and T14, respectively). Grouping of the samples was done by means of hierarchical clustering on principal components. Clusters are numbered from 1-6. Different treatments and timepoints are indicated in different colours.

To get a better understanding of overall differences in fungal and bacterial community composition across treatments and timepoints, the data were grouped by means of hierarchical clustering on principal components (HCPC), with the aim to identify groups with similar observations in the dataset. Six clusters were suggested and were hallmarked by specific phylogenetic classes per cluster (**Figure 4**). Firstly, cluster 1 comprised of solely two samples, both *F. graminearum*-infected, presymptomatic samples taken at 7 dpi. The samples within this cluster were significantly dominated by Acidimicrobiia, Acidobacteriae, Clostridia, Thermoleophilia and Malasseziomycetes (p = 0.007, < 0.001, < 0.001, < 0.001 and 0.030, respectively). Secondly, cluster 2 constituted of five blank samples which were significantly more abundant in Gammaproteobacteria, Cystobasidiomycetes, Malasseziomycetes, Microbotryomycetes, Mortierellomycetes and Tremellomycetes compared to the overall mean relative abundance (p = 0.002, 0.001, 0.042, 0.027, < 0.001 and < 0.001, respectively). Thirdly, both samples of cluster 3 were presymptomatic, infected control samples taken at 7 dpi and were more dominant in Acidimicrobiia, Bacteroidia, Parcubacteria and Verrucomicrobiae (p = 0.041, < 0.001, < 0.001 and < 0.001, respectively). Next, cluster 4 was the biggest cluster consisting of 20 samples, all significantly higher abundant in Leotiomycetes when compared to the overall mean relative abundance (p = 0.001). In cluster 5, mainly non-infected samples were observed, although one symptomless infected control was incorporated in this cluster as well. Samples within cluster 5 were more abundant in Alphaproteobacteria and Dothideomycetes whereas Gammaproteobacteria, Leotiomycetes, Malasseziomycetes, Planctomycetes and Sordariomycetes were significantly less represented when compared to the other clusters (p = < 0.001, < 0.001, < 0.001, < 0.001, 0.028, 0.026 and 0.046, respectively). Lastly, samples within cluster 6 were dominated by members of Planctomycetes, Saccharimonadia, Vicinamibacteria and Sordariomycetes, while Dothideomycetes, Leotiomycetes and Ustilaginomycetes were less abundant in this cluster (p = 0.001, 0.003, 0.046, < 0.001, 0.007, 0.001 and 0.019, respectively). Most samples within this cluster were infected with *F. graminearum* and showed FHB symptoms, causing the elevated abundance of Sordariomycetes as demonstrated earlier (**Figure 3**). However, one sample within cluster 6 was not infected with *F. graminearum* but solely inoculated with *S. rimosus* subsp. *rimosus* LMG19352 and showed a different abundance pattern than the other samples in this cluster.

Overall, some general trends could be observed from the HCPC. On the one hand, cluster 1, 2 and 3 were completely covered by non-symptomatic wheat ears sampled at 0 or 7 dpi. On the other hand, cluster 6 mainly comprised of infected, symptomatic samples taken at 14 dpi. Cluster 4 and 5 were positioned in the center and formed a blend between all treatments and sampling timepoints, except for the infected control samples at 14 dpi which were solely present in cluster 6. From these results, it can be suggested that cluster formation was not completely based on treatment or sampling timepoint although they both gave the impression to have an influence as the infection with *F. graminearum* and more specifically the induction of FHB symptoms seemed to have an effect on grouping the samples into clusters. Therefore, FHB symptoms are suggested to play a role in shaping the spicosphere microbiome.

### 3.4 Topological network parameters reveal that correlation networks differ in complexity and stability based on treatment

Cross-kingdom correlation networks were created for each treatment at each sampling timepoint (**Supplementary Figures 4**-**12**). Multiple topological parameters of the correlation networks (**Table 4**) revealed that they differ among treatments in complexity and stability.

**Table 4:** Topological parameters of microbial correlation networks of uninoculated wheat ears (blank), wheat ears inoculated with *F. graminearum* (Fg control), wheat ears inoculated with *S. rimosus* subsp. *rimosus* LMG19352 (LMG19352) and wheat ears co-inoculated with *F. graminearum* and *S. rimosus* subsp. *rimosus* LMG19352 (Fg+LMG19352) at 0, 7 and 14 days post infection (DPI).

		Blank	Fg control	LMG19352	Fg+LMG19352
Nodes	0 DPI	24	24	24	24
	7 DPI	20	24	22	21
	14 DPI	20	17	23	24
Edges	0 DPI	44	44	44	44
	7 DPI	49	53	83	41
	14 DPI	52	32	60	84
Positive edges (%)	0 DPI	77	77	77	77
	7 DPI	18	34	37	41
	14 DPI	31	25	30	29
Negative edges (%)	0 DPI	23	23	23	23
	7 DPI	82	66	63	59
	14 DPI	69	75	70	71
Degree	0 DPI	3.66	3.66	3.66	3.66
	7 DPI	4.90	4.42	7.54	3.90
	14 DPI	5.20	3.76	5.22	7.00
Density	0 DPI	0.08	0.08	0.08	0.08
	7 DPI	0.13	0.10	0.18	0.10
	14 DPI	0.14	0.12	0.12	0.15
Clustering	0 DPI	0.66	0.66	0.66	0.66
	7 DPI	0.51	0.64	0.73	0.64
	14 DPI	0.61	0.82	0.75	0.63
Assortativity	0 DPI	0.06	0.06	0.06	0.06
	7 DPI	0.34	0.53	0.30	0.18
	14 DPI	-0.12	0.82	0.55	0.09
Modularity	0 DPI	0.46	0.46	0.46	0.46
	7 DPI	0.35	0.46	0.27	0.41
	14 DPI	0.35	0.41	0.36	0.28
Shortest path length	0 DPI	1.84	1.84	1.84	1.84
	7 DPI	2.27	3.07	1.94	2.43
	14 DPI	1.79	1.14	1.53	1.88

The complexity of the networks was evaluated based on the number of nodes and edges, together with the mean degree and density. The number of nodes were relatively stable throughout the treatments and throughout time, confirming a stable microbial diversity as revealed earlier (**Table 3**). However, a decrease in number of nodes was observed during the symptomatic stage of the infected control, which indicates that the microbial diversity decreases throughout time upon FHB symptom development. In the blank, the number of edges increased over time, indicating a more interconnected community over time. The combined inoculation of *F. graminearum* and LMG19352 showed a similar pattern to the blank, although more connections (i.e. edges) were observed at 14 dpi than in the blank control. Contrastingly, in wheat ears inoculated with *Fusarium* alone, the number of edges first increased and subsequently decreased over time, indicating a loss of complexity in the microbiome upon *Fusarium* and FHB symptom proliferation. The same pattern was observed in ears inoculated with LMG19352 alone due to the high number of edges at 7 dpi. Though, a higher number of connections than the blank control was observed at 14 dpi, which was not the case for the infected control samples. To conclude, besides the high number of edges in the correlation networks of treatment LMG19352 at 7 dpi and Fg+LMG19352 at 14 dpi, they also had a higher degree and density, suggesting those two networks are more complex when compared to the rest, while the correlation network of the infected control showed a lower complexity at the symptomatic stage.

Further analysis revealed that the correlation networks at 14 dpi of the infected control, LMG19352 and Fg+LMG19352 had a lower proportion of positive edges compared to the blank control. The lowest value was measured for the infected control, indicating the strongest competition among classes. Additionally, the clustering coefficient and assortativity were higher in the infected control at 14 dpi than in all other treatments while on the other hand, average path length was lowest. These results suggest an efficient network, i.e. a network wherein highly interconnected nodes tend to cluster together with other interconnected nodes and vice versa.

The networks of the blank control at 0 dpi, Fg control at 7 and 14 dpi, and Fg+LMG19352 at 7 dpi all had a modularity index slightly greater than 0.4, implying a modular structure of the network (Newman, 2006). Herein, nodes tend to correlate with other nodes within the same modules, but have sparse connections between nodes in different modules. Over time, the modularity tended to decrease, except for treatment LMG19352. The lowest values were observed for treatment LMG19352 at 7 dpi and Fg+LMG19352 at 14 dpi. This could indicate that interaction patterns in the network are more homogeneous, meaning there are fewer distinct groups of highly connected nodes. Instead, interactions are more evenly distributed across the network. On the other hand, low modularity in a biological network may also be a hallmark of a diseased or dysfunctional state, characterized by reduced robustness to damage or environmental stress. Though the latter hypothesis is considered unlikely, since both networks were evaluated as the most complex and previously shown results indicated a diverse and balanced microbiome for both treatments and timepoints, similar to the blank control (**Figure 3**).

**Figure 5:**
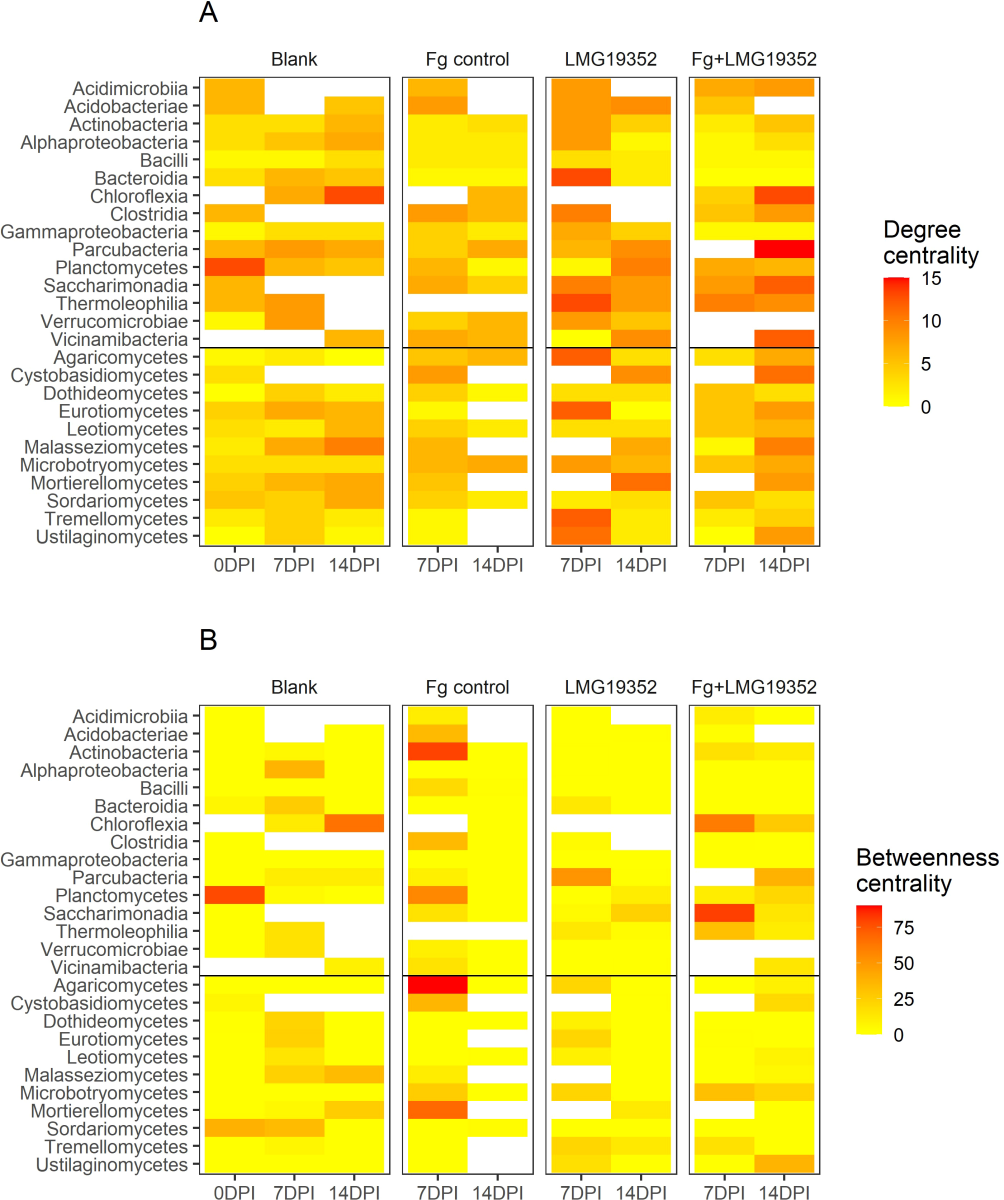
Heatmaps of degree **(A)** and betweenness centrality **(B)** of microbial correlation networks at taxonomical class-level of uninoculated wheat ears (blank), wheat ears inoculated with *F*. *graminearum* (Fg control), wheat ears inoculated with *S. rimosus* subsp. *rimosus* LMG19352 (LMG19352) and wheat ears co-inoculated with *F*. *graminearum* and *S. rimosus* subsp. *rimosus* LMG19352 (Fg+LMG19352) at 0, 7 and 14 days post infection (DPI).

Centrality measures, degree and betweenness, were used in quantitative representation of keystone species. Degree, i.e. the number of direct correlations to a node in the network, and betweenness centrality, i.e. the importance of a class as connector in the network, differ throughout sampling timepoints and across treatments (**Figure 5**). High centrality values indicate that a microbial taxon plays a more crucial role in the network structure of a certain treatment at a given timepoint.

Some bacterial and fungal taxa exhibited notable changes in degree centrality over time and across treatments (**Figure 5A**). For instance, Planctomycetes and Chloroflexia appeared to have an increased degree centrality in the blank control at 7 and 14 dpi, respectively. This suggests that these groups become more interconnected and potentially important as the microbial communities change under these conditions. In the networks of the infected control (Fg control) at 7 and 14 dpi, maximum degree was lower compared to the other treatments with maximum values of 8 and 7, respectively. For treatment LMG19352, more taxa had a degree centrality value ≥10 at 7 dpi than at 14 dpi, while for the co-inoculated wheat ears the opposite trend was observed. However, the taxa with elevated degree centrality values were different between both treatments and timepoints.

The betweenness centrality values are relatively lower than the degree centrality values, indicating that few taxa serve as connectors between groups (**Figure 5B**). Moreover, in total 37% of all betweenness centrality values was zero. However, some bacterial and fungal classes exhibited high betweenness centrality values as well. For example, a value of 78 was determined for Planctomycetes in the blank control at 0 dpi. Additionally, Saccharimonadia displayed a value ≥ 80 in the co-inoculated network at the presymptomatic stage, while in the network of the infected control at that timepoint the highest betweenness centrality values (≥ 80) were shown for Actinobacteria and Agaricomycetes. These findings indicate that all these taxa act as key connectors within the microbial network of different treatments and different timepoints. Contrastingly, only low betweenness centrality values (≤ 4) were measured for all taxa in the network of the infected control during the symptomatic stage. However, it needs to be mentioned that more than half of the fungal classes did not show any significant correlations with other classes and thus, were excluded from the network.

When comparing both centrality measures, we were able to find three potential hub taxa out of three different networks. Over all treatments and timepoints, the highest degree was observed in the network of symptomatic, co-inoculated samples with a value of 15 for Parcubacteria. Within this network, the highest value for betweenness centrality was determined for Parcubacteria as well. These results imply their function as hub taxa within this network. Secondly, Planctomycetes stood out as potentially influential bacterial taxa in the blank control at 0 dpi, bridging the bacterial cluster with solely positive connections to the rest of the network (**Supplementary Figure 4**). Thirdly, Chloroflexia acted as an important hub node in the blank control network at 14 dpi, having the highest degree and betweenness centrality values within that network. In all three networks, the potential hub taxa are located in the center of the network (**Supplementary Figures 4**, **6**, **12**), confirming their important role in maintaining the stability of the network structure.

## 4 Discussion

The use of BCAs to control phytopathogens, such as *F. graminearum* in wheat, offers a promising and sustainable alternative to chemical fungicides. In this study, we first evaluated the biocontrol potential of *S. rimosus* subsp. *rimosus* LMG19352 on wheat ears through multispectral imaging. However, when applying a BCA to the field it is crucial to acknowledge its non-target impact on the endogenous microbiome and preferable to maintain a diverse and balanced microbiome as this could affect plant health (Alukumbura et al., 2022; Foo et al., 2017). Therefore, we studied the effects of the application of BCA *S. rimosus* subsp. *rimosus* LMG19352 and/or *F. graminearum* over time and across treatments on both the fungal and bacterial spicosphere community structure and composition, employing Illumina MiSeq. Furthermore, correlation network analyses were performed across all different conditions (treatment and timepoint) to explore the complexity and stability of these spicosphere microbiomes.

### 4.1 Mycotoxin production might influence shifts in the fungal spicosphere microbiome upon *Fusarium graminearum* colonization and Fusarium Head Blight symptom development

The symptomatic stage of FHB resulted in a significantly lower α-diversity in fungal communities from *F. graminearum*-treated wheat ears compared to nontreated ones, whereas the α-diversity in bacterial communities was not significantly altered. Additionally, topological and centrality features of the correlation network elicited a less complex network at the symptomatic stage of *F. graminearum*-infected wheat ears. Both a reduced α-diversity and a less complex network indicate a diseased status of the microbiome, as previously shown in other studies (Cardoni et al., 2023; Legrand et al., 2019; Rojas et al., 2020). In addition, when comparing flowering stage (i.e. 0 dpi) with mid-grain filling stage (i.e. 14 dpi) of *F. graminearum* inoculated ears, multiple significant shifts in the fungal spicosphere microbiome community were determined, whereas it was not at early grain filling stage (i.e. 7 dpi). Similarly, some significant changes were observed when comparing blank and infected control ears at mid-grain filling stage (i.e. 14 dpi) but not at early grain filling stage (i.e. 7 dpi). These results indicate that shifts in the fungal spicosphere microbiome are not directly caused by the inoculation with *F. graminearum* itself but rather by the development of FHB symptoms. These shifts may be triggered by increased production of mycotoxins such as deoxynivalenol (DON), which becomes apparent during symptom development as the mycotoxin is crucial for the spread and colonization of the fungus within the wheat rachis (Audenaert et al., 2014). DON interacts specifically with eukaryotic systems by forming three hydrogen bonds with the A-site of the 60S ribosomal subunit, inhibiting peptidyl transferase activity. This specificity could explain why the bacterial microbiome in the wheat spicosphere remained more stable than the fungal microbiome. Our hypothesis aligns with findings from Bakker and McCormick (2019), who reported a negative correlation between DON levels and microbial richness, evenness, and diversity, with more pronounced effects on fungal communities. However, they concluded that the microbiome did not independently influenced toxin accumulation beyond its effects on pathogen load. Future studies could elicit the impact of DON on shifts in the spicosphere microbiome by performing an additional mycotoxin analysis or by including wheat ears inoculated with DON.

### 4.2 Inoculation of wheat ears with biocontrol agent *Streptomyces rimosus* subsp. *rimosus* LMG19352 causes limited long-term off-target effects on the spicosphere microbiome in the absence of *Fusarium graminearum*

At early grain filling stage (i.e. 7 dpi), significant changes in the spicosphere structure and composition of fungal communities were observed after applying *S. rimosus* subsp. *rimosus* LMG19352. The BCA-inoculated wheat ears showed a significant decrease in fungal richness, evenness and diversity when compared to the blank control. This reduction could largely be attributed to a high number of counts for a specific fungal genus, *Cladosporium* (class of Dothideomycetes), which is naturally present on wheat ears, as confirmed by its consistent isolation across sampling sites in a study conducted in Germany (Schiro et al., 2019). This fungal genus may have benefited from the inoculation with *S. rimosus* subsp. *rimosus* LMG19352 and outcompeted other fungal genera, resulting in a reduced richness, evenness and diversity. These results were supported by a significant negative correlation of Dothideomycetes with both Sordariomycetes and Leotiomycetes, as displayed in the correlation network. Similarly, Rojas et al. (2020) found that *Cladosporium* (class of Dothideomycetes) negatively correlated with *Fusarium* spp. (class of Sordariomycetes) on wheat ears, linking it to spikes that remained healthy (Rojas et al., 2020). Although *Cladosporium herbarum* is known to cause Cladosporium Ear Rot disease on maize, some other species have been explored as mycoparasitic BCAs on wheat against several fungal diseases such as *Blumeria graminis* (Zhu et al., 2024) and *Puccinia striiformis* f. sp. *tritici* (Zhang et al., 2022). Notably, in wheat ears treated with *S. rimosus* subsp. *rimosus* LMG19352 alone, the relative abundance of *Blumeria* was lower, suggesting that Cladosporium’s increased abundance may have contributed to its biocontrol activity and that the application of our BCA had an indirect effect on the biocontrol of *Blumeria*, although the pathogenic species was still omnipresent without causing disease symptoms. In future research, a higher-resolution technique with sequence information of longer reads, able to identify taxa at species or even strain level, could be used to determine which species of *Cladosporium* was observed (Knief, 2014).

At mid-grain filling stage (i.e. 14 dpi), most shifts in the fungal spicosphere microbiome were partially restored towards its original state at the non-inoculated stage (i.e. 0 dpi). Additionally, when comparing blank and LMG19352-treated wheat ears at mid-grain filling stage (i.e. 14 dpi), no significant differences could be observed between both treatments. These results imply limited long-term effects on non-target microorganisms after applying BCA *S. rimosus* subsp. *rimosus* LMG19352 on wheat ears in the absence of a *F. graminearum* infection. These results can provide a starting point for extended greenhouse trials or field trials by means of validation.

### 4.3 Co-inoculation of wheat ears with *Streptomyces rimosus* subsp. *rimosus* LMG19352 and *Fusarium graminearum* results in effective biocontrol and minimal off-target effects on the spicosphere microbiome

Co-inoculating wheat ears with BCA *S. rimosus* subsp. *rimosus* LMG19352 and *F. graminearum* resulted in a significant decrease of FHB symptoms on infected spikelets during the symptomatic stage when compared to the infected control. These results are in agreement with earlier findings of Tan et al. (2021), who attributed this inhibitory effect to direct antagonism. Additionally, the fungal and bacterial community structures of co-inoculated ears were not significantly different from both the infected or blank control but acted intermediate, similar to findings of Alukumbura et al. (2022) who inoculated wheat ears with *T. gamsii* T6085 as BCA against naturally occurring FHB and could also not distinguish significant differences in fungal and bacterial community structures between BCA-treated and infected control plants. In addition, the fungal relative abundances of co-inoculated ears remained more stable over time when compared to the infected control ears. However, the relative abundance of Sordariomycetes (class of *F. graminearum*) in co-inoculated ears was intermediate and not significantly different from the blank and infected control, which is in accordance with F_v_/F_m_ and cGFP values of the sampled spikelets and results of the HCPC. A plausible explanation for the intermediate effect on *F. graminearum* inhibition is the variable efficacy of the BCA, as often reported as a challenge for other BCAs. Combining BCAs among themselves or with chemical fungicides could help to reduce their variability and inconsistency (Ons et al., 2020; Xu et al., 2011). Alternatively, fluctuations in the microbiome could have influenced the BCA efficacy as well. For example, the efficacy of a BCA could be enhanced by the presence of a helper strain in the endogenous microbiome – an organism which does not have biocontrol properties itself but facilitates or enhances biocontrol properties of the BCA through, e.g. better BCA establishment, enhanced survival on the host, or improved metabolite production (Massart et al., 2015). In our study, Leotiomycetes might have acted as such a helper class since their relative abundance was significantly lower in cluster 6 of the HCPC, which includes samples with the highest abundances of Sordariomycetes. Additionally, their relative abundance was significantly increased in cluster 4 of the HCPC, containing three out of five replicates of co-inoculated ears at the symptomatic stage with reduced abundance of Sordariomycetes, potentially increasing the biocontrol potential of our BCA.

Together, these results indicate that BCA *S. rimosus* subsp. *rimosus* LMG19352 tempers shifts in the spicosphere microbiome induced by FHB symptoms, promoting a diverse and balanced microbiome with reduced abundance of *F. graminearum*. This suggests its role as BCA with a relatively novel mode of action, as previously described by Erlacher et al. (2014). Similarly, they demonstrated that the rhizosphere and phyllosphere microbiome of lettuce shifts as a consequence to pathogen attack but that BCA *Bacillus amyloliquefaciens* FZB42 can compensate these effects. Additionally, previous research has shown that *S. rimosus* subsp. *rimosus* LMG19352 is able to diminish the production of the mycotoxin DON, and to mitigate another important mycotoxin, zearalenone (ZEN), *in vitro* and *in planta* (De Troyer et al., 2024; Tan et al., 2021), supporting our hypothesis that shifts in the spicosphere microbiome are triggered by the production of mycotoxins. However, it remains unclear whether these changes are directly driven by *S. rimosus* subsp. *rimosus* LMG19352 since no elevated abundance in Actinobacteria was detected over time in the sampled spikelets of co-inoculated ears, which supports previous findings regarding the limited motility of *Streptomyces* spp (Muok et al., 2021). In future studies, tagging the inoculated bacterial strain with a fluorescent marker, e.g. RFP, could provide insights in its capacity to colonize wheat ears and could clarify whether the inoculation with *S. rimosus* subsp. *rimosus* LMG19352 directly influences the abundance of Actinobacteria in the sampled spikelets. Alternatively, applying the BCA via spraying may address this challenge of limited motility and may provide an effective alternative approach, since it has been demonstrated that *S. rimosus* subsp. *rimosus* LMG19352 could persist at the inoculated spikelet of wheat ears for at least 25 days (De Troyer et al., 2024).

Overall, the results of this study are promising, although further scientific research is required before *S. rimosus* subsp. *rimosus* LMG19352 can be implemented in the field as a BCA against FHB. Field trials are essential to fully assess the efficacy of *S. rimosus* subsp. *rimosus* LMG19352, as BCAs often show reduced performance under field conditions. For instance, Colombo et al. (2019) demonstrated that the biocontrol effect of *Streptomyces* sp. DEF09 was lower in a field study when compared to a greenhouse experiment. Future field studies should also consider incorporating more biological replicates, since within-treatment variability is likely to increase under field conditions, where environmental factors such as temperature and relative humidity cannot be controlled. Additionally, the spicosphere microbiome in field studies should be investigated as it will be more representative to the native microbiome compared to pot experiments. However, the wheat microbiome is influenced by multiple factors as reviewed by Kavamura et al. (2021), including anthropogenic factors (e.g. fertilizer use), soil properties (e.g. soil type), environmental conditions (e.g. abiotic stress), and host factors (e.g. growth stage), which complicates generalization of results across studies. Nonetheless, the results of this study together with all the features demonstrated in previous research on the biocontrol activity of *S. rimosus* subsp. *rimosus* LMG19352 against *F. graminearum* and its mycotoxins (De Troyer et al., 2024; Tan et al., 2021), make it a suitable candidate for further investigations as a BCA against FHB.

## 5 Conclusion

The application of *S. rimosus* subsp. *rimosus* LMG19352 on *F. graminearum*-infected wheat ears confirmed its biocontrol effect by reducing *F. graminearum* growth and preventing disease proliferation. Additionally, analysis of the spicosphere microbiome revealed that shifts in fungal community composition, induced by FHB symptoms, are tempered towards a more diverse and balanced microbiome upon application of the BCA. These results indicate minimal off-target effects of the use of biocontrol agent *S. rimosus* subsp. *rimosus* LMG19352 and make it a suitable candidate for subsequent field trials in the quest for the sustainable control of FHB.

## Data Availability

The datasets presented in this study can be found in online repositories. The names of the repository/repositories and accession number(s) can be found below: https://www.ncbi.nlm.nih.gov/, PRJNA1193851.

## Supplementary material

Supplementary Figure 1 Spearman correlation matrix between α-diversity measures (chao1 richness, Pielou’s evenness and Shannon diversity) of fungal (FUN) and bacterial (BAC) communities, and important plant parameters (plant health (FvFm) and *F. graminearum* growth (cGFP)) across all timepoints (0, 7 and 14 days post infection) and treatments (uninoculated wheat ears (blank), wheat ears inoculated with *F. graminearum* (Fg control), wheat ears inoculated with *S. rimosus* subsp. *rimosus* LMG19352 (LMG19352) and wheat ears co-inoculated with *F. graminearum* and *S. rimosus* subsp. *rimosus* LMG19352 (Fg+LMG19352)). Index *i* and *s* indicate plant parameters were measured at the inoculated spikelet and sampled spikelet, respectively.

Supplementary Figure 2 Venn diagram for the number of shared and unique bacterial and fungal ASVs of uninoculated wheat ears (blank), wheat ears inoculated with *F. graminearum* (Fg control), wheat ears inoculated with *S. rimosus* subsp. *rimosus* LMG19352 (LMG19352) and wheat ears co-inoculated with *F. graminearum* and *S. rimosus* subsp. *rimosus* LMG19352 (Fg+LMG19352) at 0, 7 and 14 days post inoculation (DPI), represented under different timepoints across all treatments **(A)** and under different treatments across all timepoints **(B)**.

Supplementary Figure 3 Relative abundances (%) of bacterial classes of uninoculated wheat ears (blank), wheat ears inoculated with *F. graminearum* (Fg control), wheat ears inoculated with *S. rimosus* subsp. *rimosus* LMG19352 (LMG19352) and wheat ears co-inoculated with *F. graminearum* and *S. rimosus* subsp. *rimosus* LMG19352 (Fg+LMG19352) at 0, 7 and 14 days post inoculation (DPI) with no significant differences between timepoints or treatments. Values represent means ± standard deviation of 5 replicates.

Supplementary Figure 4 Correlation network of blank wheat ears at 0 days post inoculation at the taxonomical level of class. Edges represent positive (blue) or negative (red) correlations. The thickness of the edges is related to the absolute value of the correlation coefficient. Nodes represent fungal (orange) or bacterial (grey) dots. The size of each node is related to the relative abundance of the respective class (n = 5 biological replicates).

Supplementary Figure 5 Correlation network of blank wheat ears at 7 days post inoculation at the taxonomical level of class. Edges represent positive (blue) or negative (red) correlations. The thickness of the edges is related to the absolute value of the correlation coefficient. Nodes represent fungal (orange) or bacterial (grey) dots. The size of each node is related to the relative abundance of the respective class (n = 5 biological replicates).

Supplementary Figure 6 Correlation network of blank wheat ears at 14 days post inoculation at the taxonomical level of class. Edges represent positive (blue) or negative (red) correlations. The thickness of the edges is related to the absolute value of the correlation coefficient. Nodes represent fungal (orange) or bacterial (grey) dots. The size of each node is related to the relative abundance of the respective class (n = 5 biological replicates).

Supplementary Figure 7 Correlation network of wheat ears inoculated with *F. graminearum* at 7 days post inoculation at the taxonomical level of class. Edges represent positive (blue) or negative (red) correlations. The thickness of the edges is related to the absolute value of the correlation coefficient. Nodes represent fungal (orange) or bacterial (grey) dots. The size of each node is related to the relative abundance of the respective class (n = 5 biological replicates).

Supplementary Figure 8 Correlation network of wheat ears inoculated with *F. graminearum* at 14 days post inoculation at the taxonomical level of class. Edges represent positive (blue) or negative (red) correlations. The thickness of the edges is related to the absolute value of the correlation coefficient. Nodes represent fungal (orange) or bacterial (grey) dots. The size of each node is related to the relative abundance of the respective class (n = 5 biological replicates).

Supplementary Figure 9 Correlation network of wheat ears inoculated with *S. rimosus* subsp. *rimosus* LMG19352 at 7 days post inoculation at the taxonomical level of class. Edges represent positive (blue) or negative (red) correlations. The thickness of the edges is related to the absolute value of the correlation coefficient. Nodes represent fungal (orange) or bacterial (grey) dots. The size of each node is related to the relative abundance of the respective class (n = 5 biological replicates).

Supplementary Figure 10 Correlation network of wheat ears inoculated with *S. rimosus* subsp. *rimosus* LMG19352 at 14 days post inoculation at the taxonomical level of class. Edges represent positive (blue) or negative (red) correlations. The thickness of the edges is related to the absolute value of the correlation coefficient. Nodes represent fungal (orange) or bacterial (grey) dots. The size of each node is related to the relative abundance of the respective class (n = 5 biological replicates).

Supplementary Figure 11 Correlation network of wheat ears co-inoculated with *S. rimosus* subsp. *rimosus* LMG19352 and *F. graminearum* at 7 days post inoculation at the taxonomical level of class. Edges represent positive (blue) or negative (red) correlations. The thickness of the edges is related to the absolute value of the correlation coefficient. Nodes represent fungal (orange) or bacterial (grey) dots. The size of each node is related to the relative abundance of the respective class (n = 5 biological replicates).

Supplementary Figure 12 Correlation network of wheat ears co-inoculated with *S. rimosus* subsp. *rimosus* LMG19352 and *F. graminearum* at 14 days post inoculation at the taxonomical level of class. Edges represent positive (blue) or negative (red) correlations. The thickness of the edges is related to the absolute value of the correlation coefficient. Nodes represent fungal (orange) or bacterial (grey) dots. The size of each node is related to the relative abundance of the respective class (n = 5 biological replicates).
