# Supplementary figures and images for: The biocontrol agent Streptomyces rimosus subsp. rimosus tempers shifts in the wheat spicosphere microbiome induced by Fusarium Head Blight
Source: Front Plant Sci. 2025 Feb 20;16:1540242. doi: 10.3389/fpls.2025.1540242 (PMC11882881; doi:10.3389/fpls.2025.1540242)

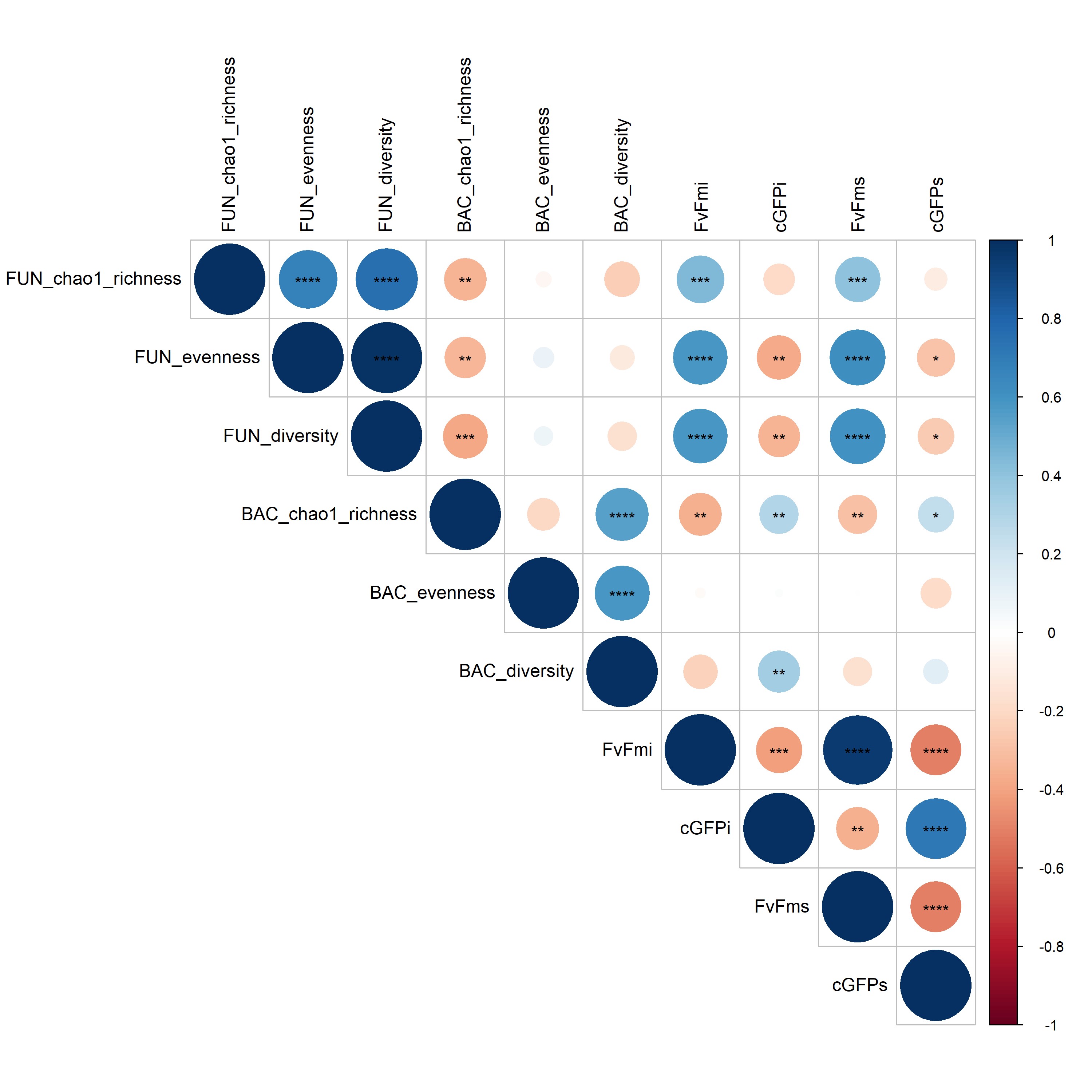

Supplement: Supplementary Figure 1 — Spearman correlation matrix between α-diversity measures (chao1 richness, Pielou’s evenness and Shannon diversity) of fungal (FUN) and bacterial (BAC) communities, and important plant parameters (plant health (FvFm) and F. graminearum growth (cGFP)) across all timepoints (0, 7 and 14 days post infection) and treatments (uninoculated wheat ears (blank), wheat ears inoculated with F. graminearum (Fg control), wheat ears inoculated with S. rimosus subsp. rimosus LMG19352 (LMG19352) and wheat ears co-inoculated with F. graminearum and S. rimosus subsp. rimosus LMG19352 (Fg+LMG19352)). Index i and s indicate plant parameters were measured at the inoculated spikelet and sampled spikelet, respectively. [file Image1.jpeg]

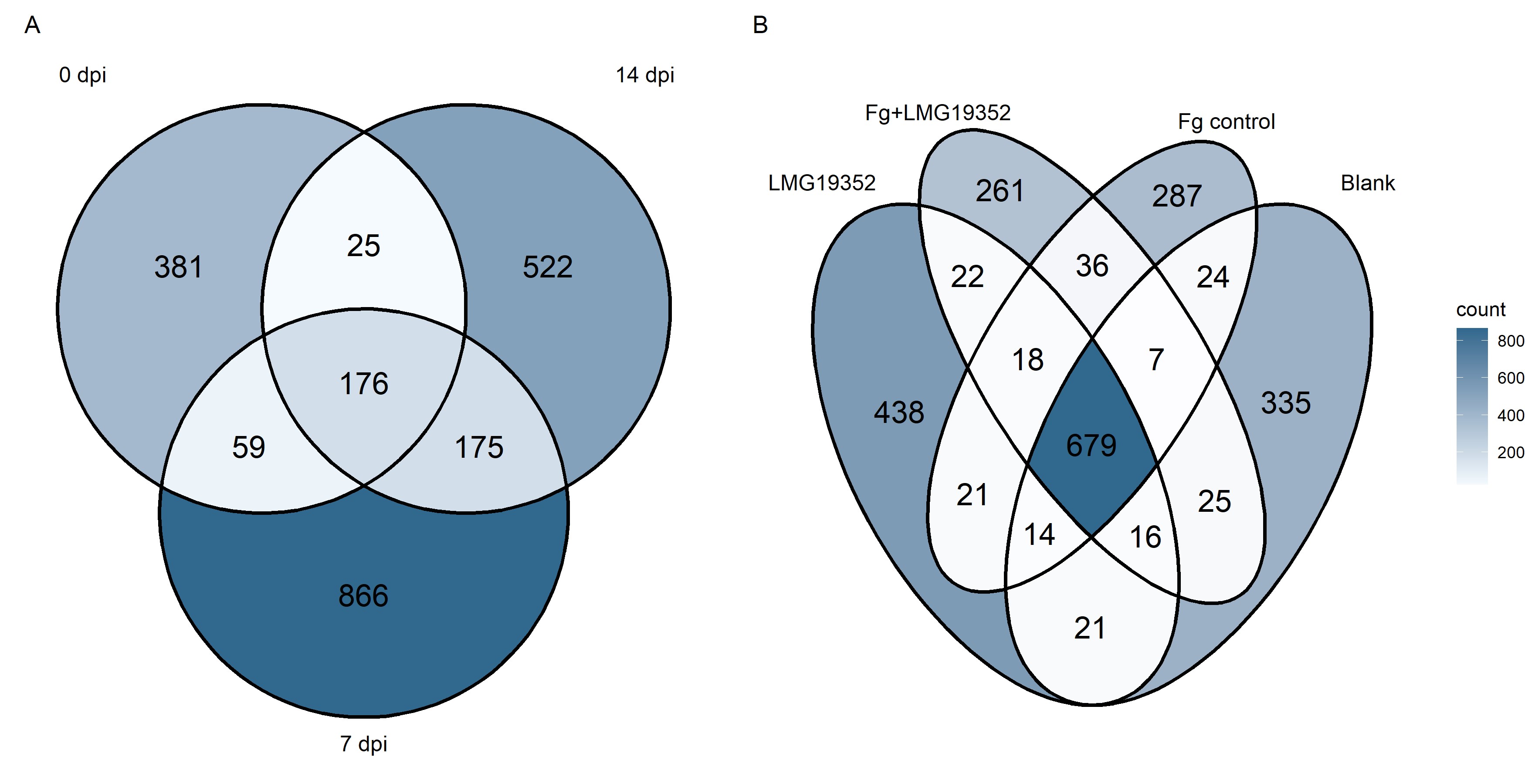

Supplement: Supplementary Figure 2 — Venn diagram for the number of shared and unique bacterial and fungal ASVs of uninoculated wheat ears (blank), wheat ears inoculated with F. graminearum (Fg control), wheat ears inoculated with S. rimosus subsp. rimosus LMG19352 (LMG19352) and wheat ears co-inoculated with F. graminearum and S. rimosus subsp. rimosus LMG19352 (Fg+LMG19352) at 0, 7 and 14 days post inoculation (DPI), represented under different timepoints across all treatments (A) and under different treatments across all timepoints (B). [file Image2.jpeg]

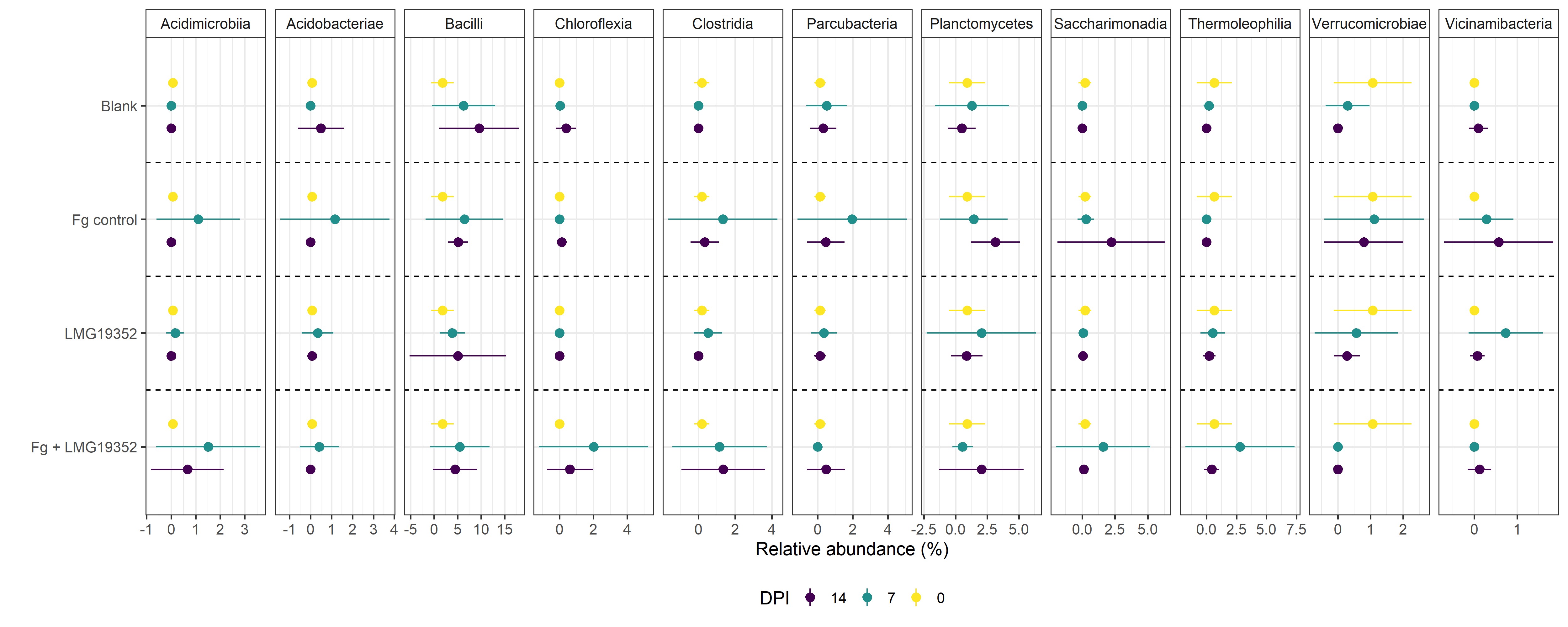

Supplement: Supplementary Figure 3 — Relative abundances (%) of bacterial classes of uninoculated wheat ears (blank), wheat ears inoculated with F. graminearum (Fg control), wheat ears inoculated with S. rimosus subsp. rimosus LMG19352 (LMG19352) and wheat ears co-inoculated with F. graminearum and S. rimosus subsp. rimosus LMG19352 (Fg+LMG19352) at 0, 7 and 14 days post inoculation (DPI) with no significant differences between timepoints or treatments. Values represent means ± standard deviation of 5 replicates. [file Image3.jpeg]

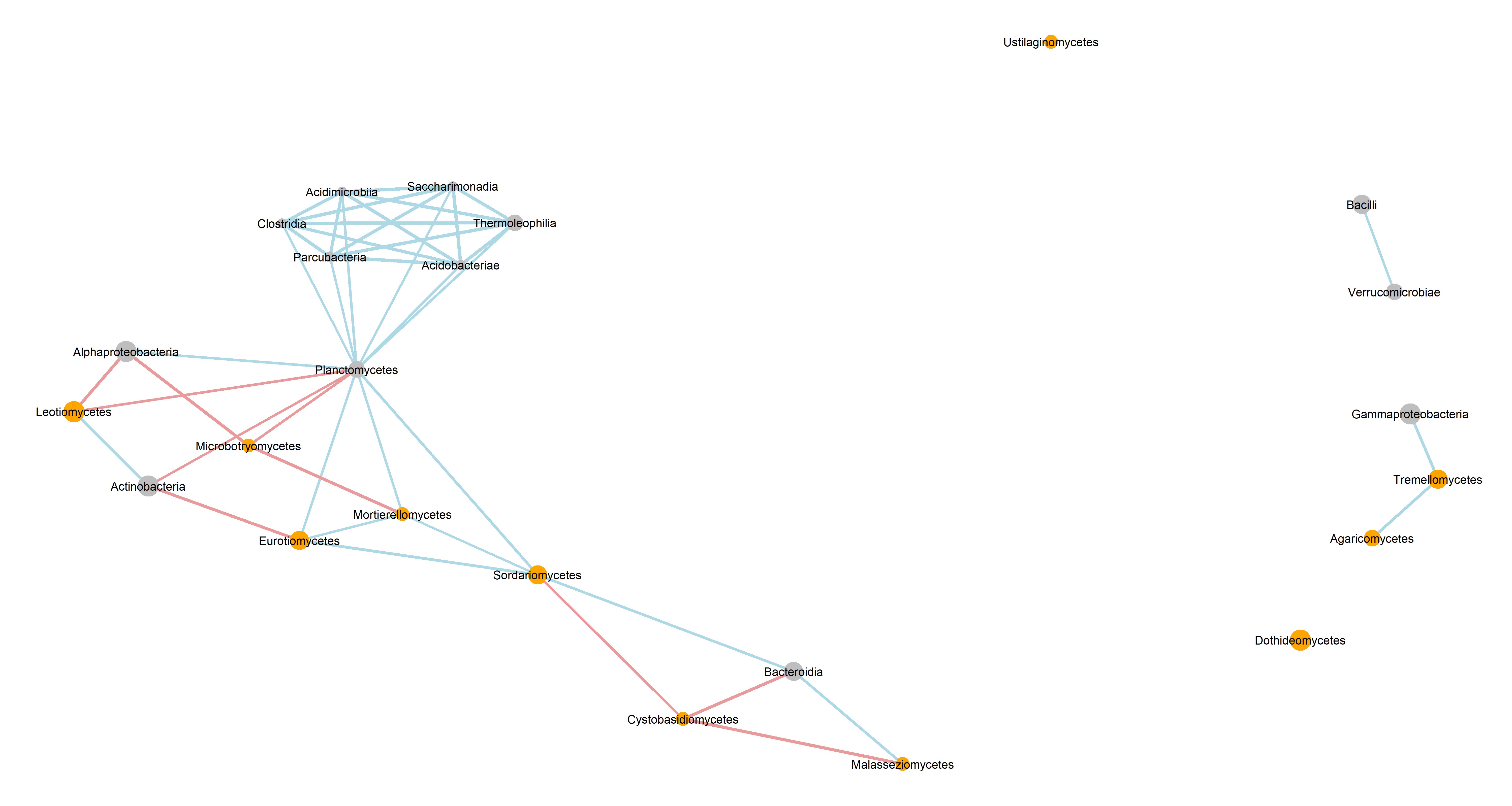

Supplement: Supplementary Figure 4 — Correlation network of blank wheat ears at 0 days post inoculation at the taxonomical level of class. Edges represent positive (blue) or negative (red) correlations. The thickness of the edges is related to the absolute value of the correlation coefficient. Nodes represent fungal (orange) or bacterial (grey) dots. The size of each node is related to the relative abundance of the respective class (n = 5 biological replicates). [file Image4.jpeg]

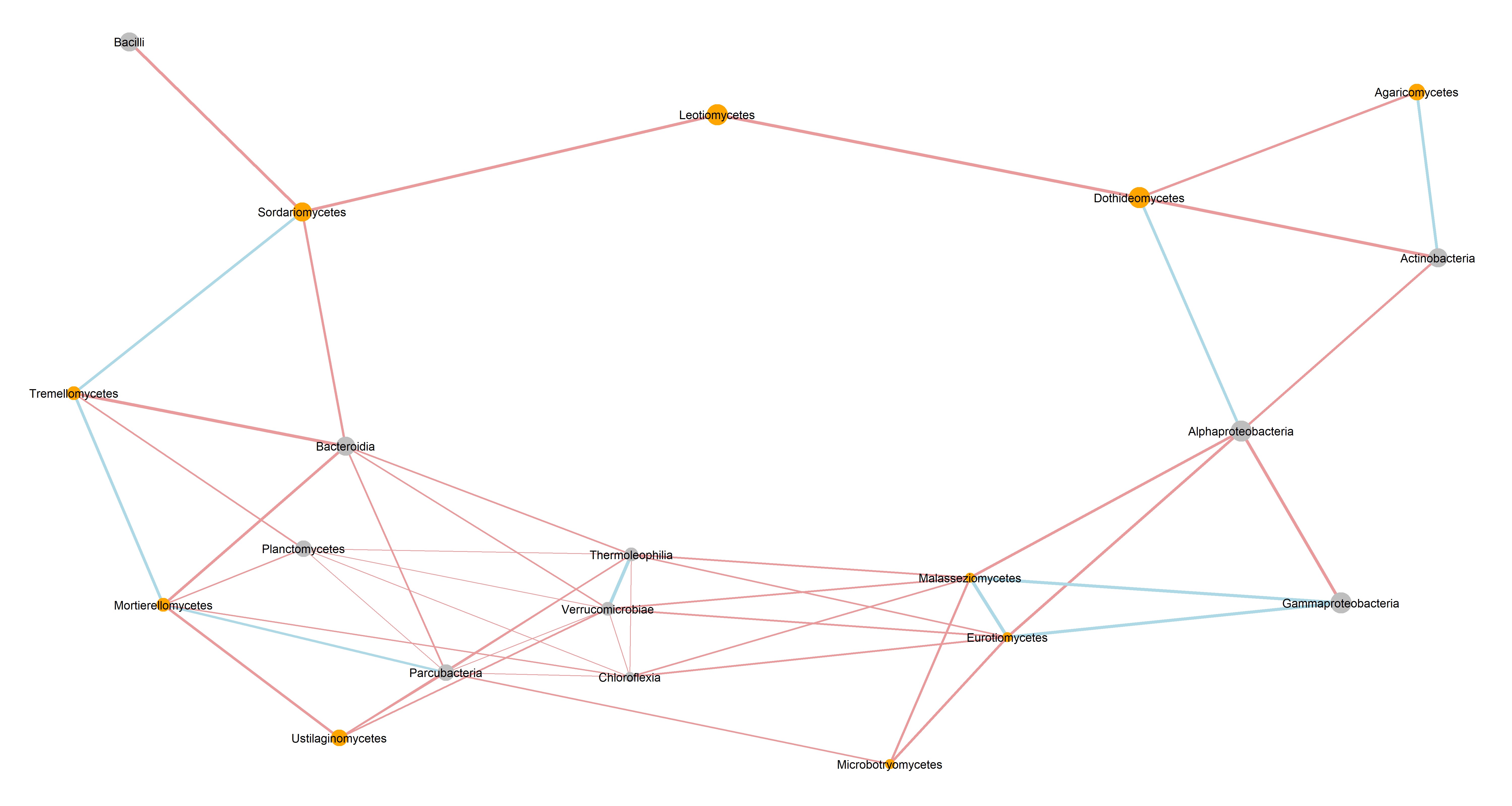

Supplement: Supplementary Figure 5 — Correlation network of blank wheat ears at 7 days post inoculation at the taxonomical level of class. Edges represent positive (blue) or negative (red) correlations. The thickness of the edges is related to the absolute value of the correlation coefficient. Nodes represent fungal (orange) or bacterial (grey) dots. The size of each node is related to the relative abundance of the respective class (n = 5 biological replicates). [file Image5.jpeg]

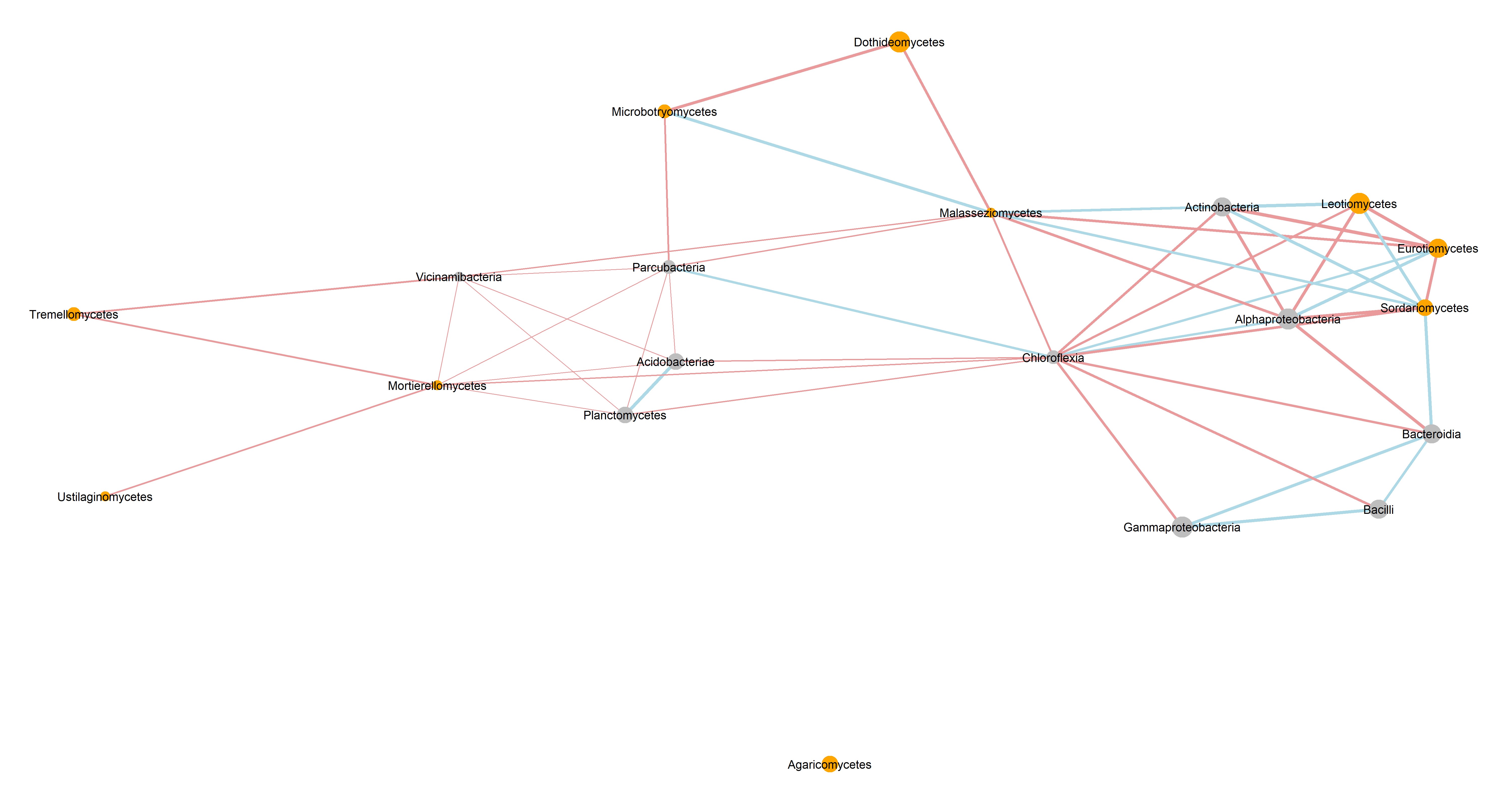

Supplement: Supplementary Figure 6 — Correlation network of blank wheat ears at 14 days post inoculation at the taxonomical level of class. Edges represent positive (blue) or negative (red) correlations. The thickness of the edges is related to the absolute value of the correlation coefficient. Nodes represent fungal (orange) or bacterial (grey) dots. The size of each node is related to the relative abundance of the respective class (n = 5 biological replicates). [file Image6.jpeg]

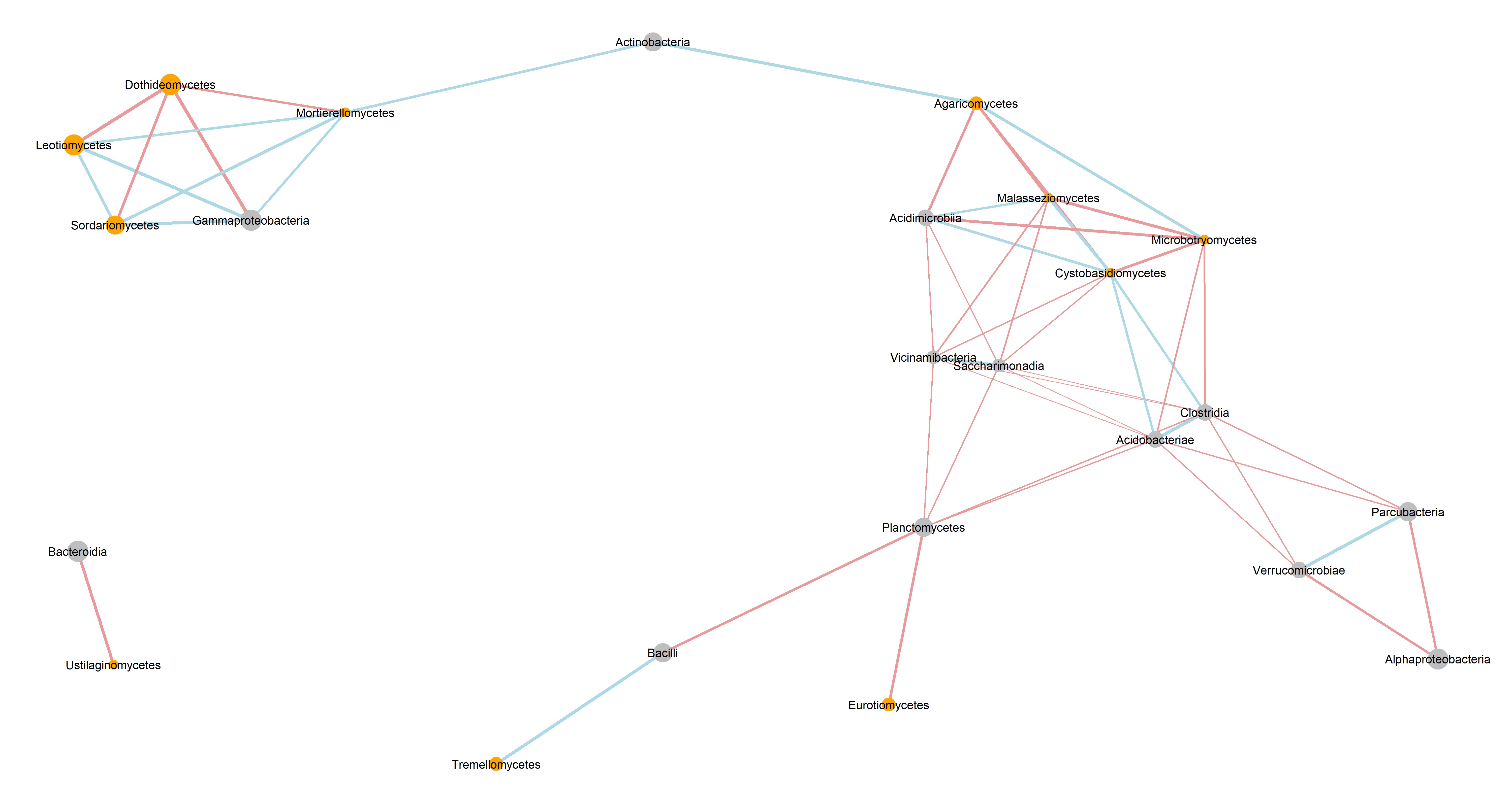

Supplement: Supplementary Figure 7 — Correlation network of wheat ears inoculated with F. graminearum at 7 days post inoculation at the taxonomical level of class. Edges represent positive (blue) or negative (red) correlations. The thickness of the edges is related to the absolute value of the correlation coefficient. Nodes represent fungal (orange) or bacterial (grey) dots. The size of each node is related to the relative abundance of the respective class (n = 5 biological replicates). [file Image7.jpeg]

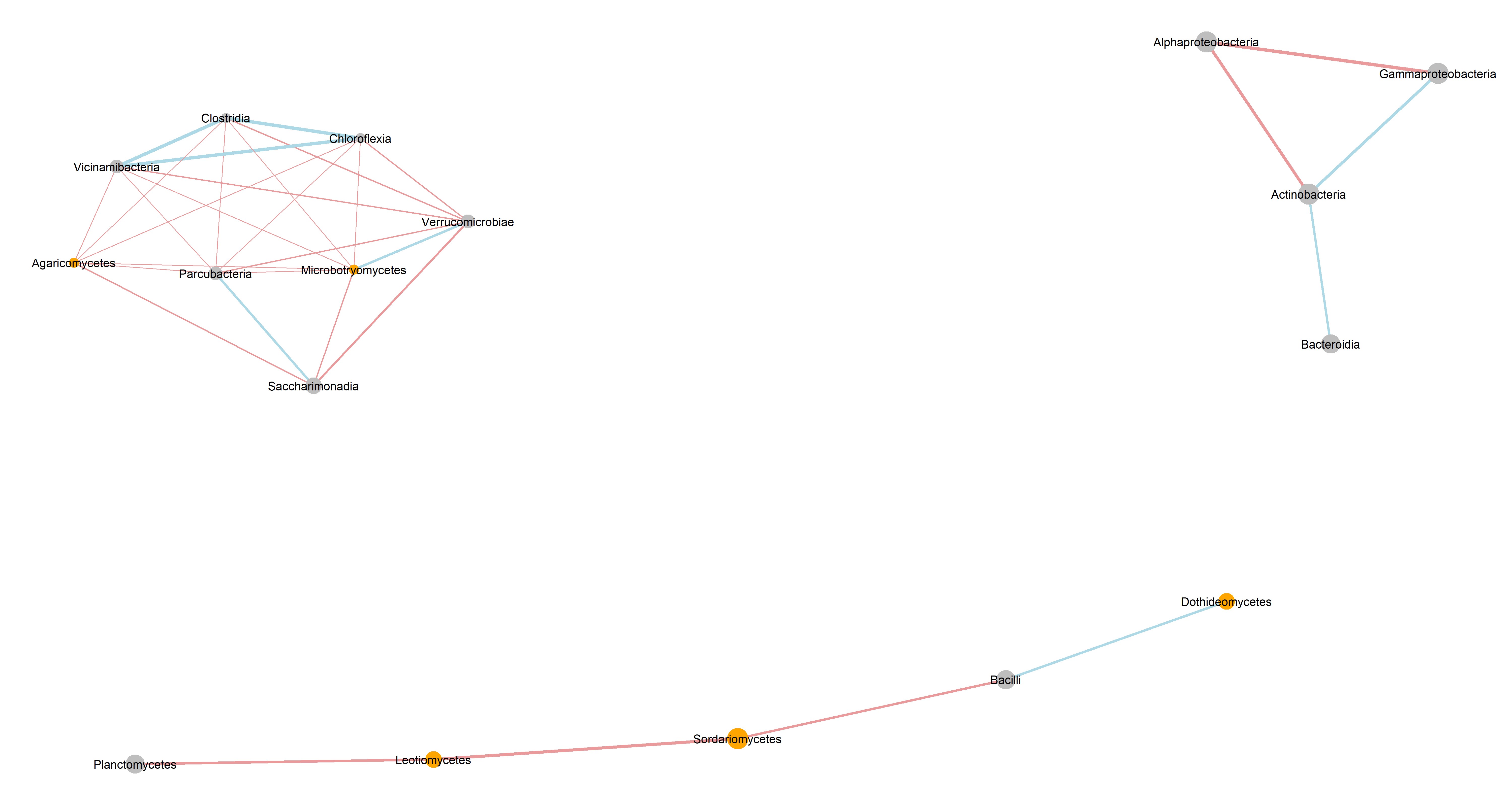

Supplement: Supplementary Figure 8 — Correlation network of wheat ears inoculated with F. graminearum at 14 days post inoculation at the taxonomical level of class. Edges represent positive (blue) or negative (red) correlations. The thickness of the edges is related to the absolute value of the correlation coefficient. Nodes represent fungal (orange) or bacterial (grey) dots. The size of each node is related to the relative abundance of the respective class (n = 5 biological replicates). [file Image8.jpeg]

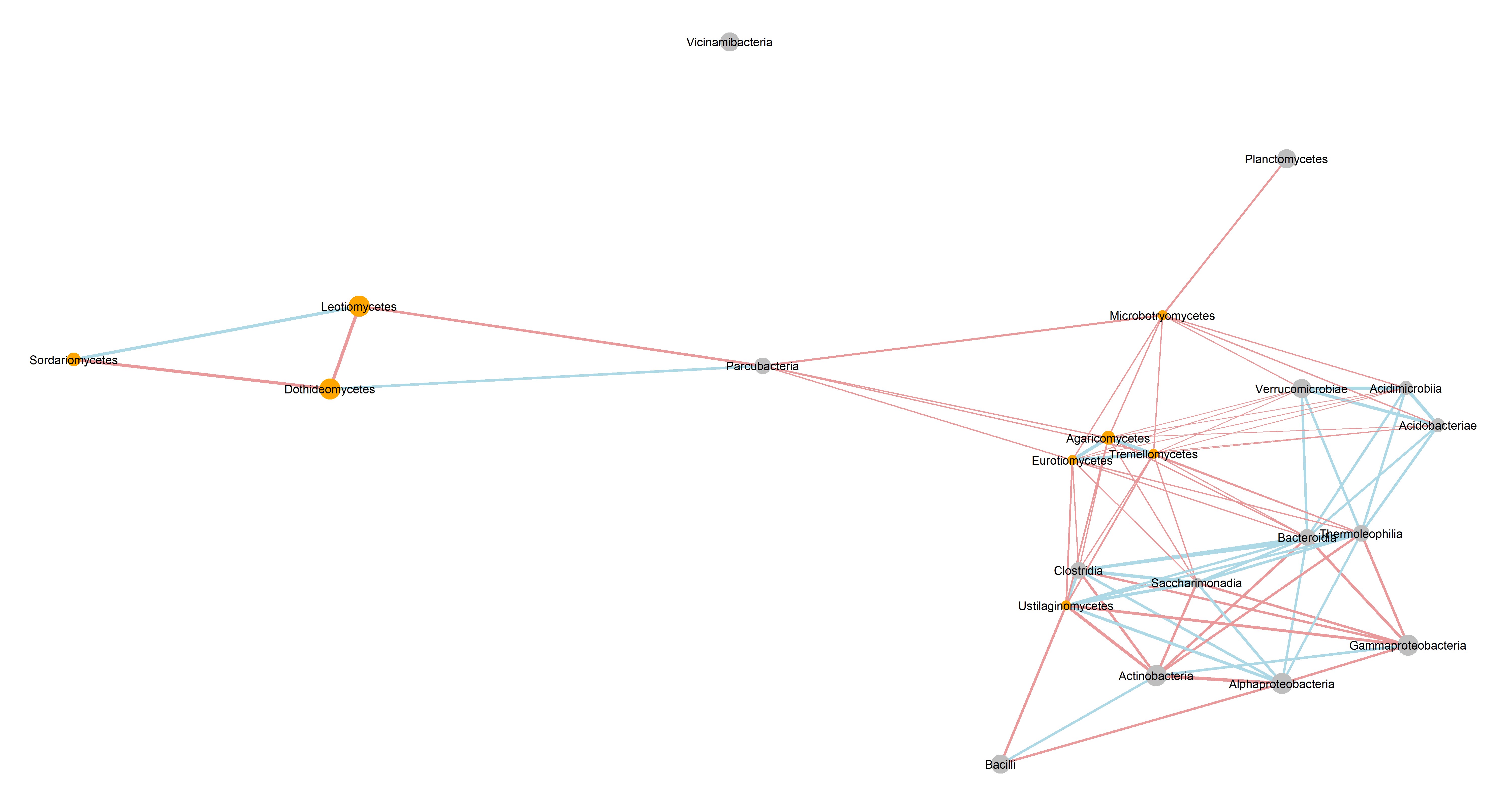

Supplement: Supplementary Figure 9 — Correlation network of wheat ears inoculated with S. rimosus subsp. rimosus LMG19352 at 7 days post inoculation at the taxonomical level of class. Edges represent positive (blue) or negative (red) correlations. The thickness of the edges is related to the absolute value of the correlation coefficient. Nodes represent fungal (orange) or bacterial (grey) dots. The size of each node is related to the relative abundance of the respective class (n = 5 biological replicates). [file Image9.jpeg]

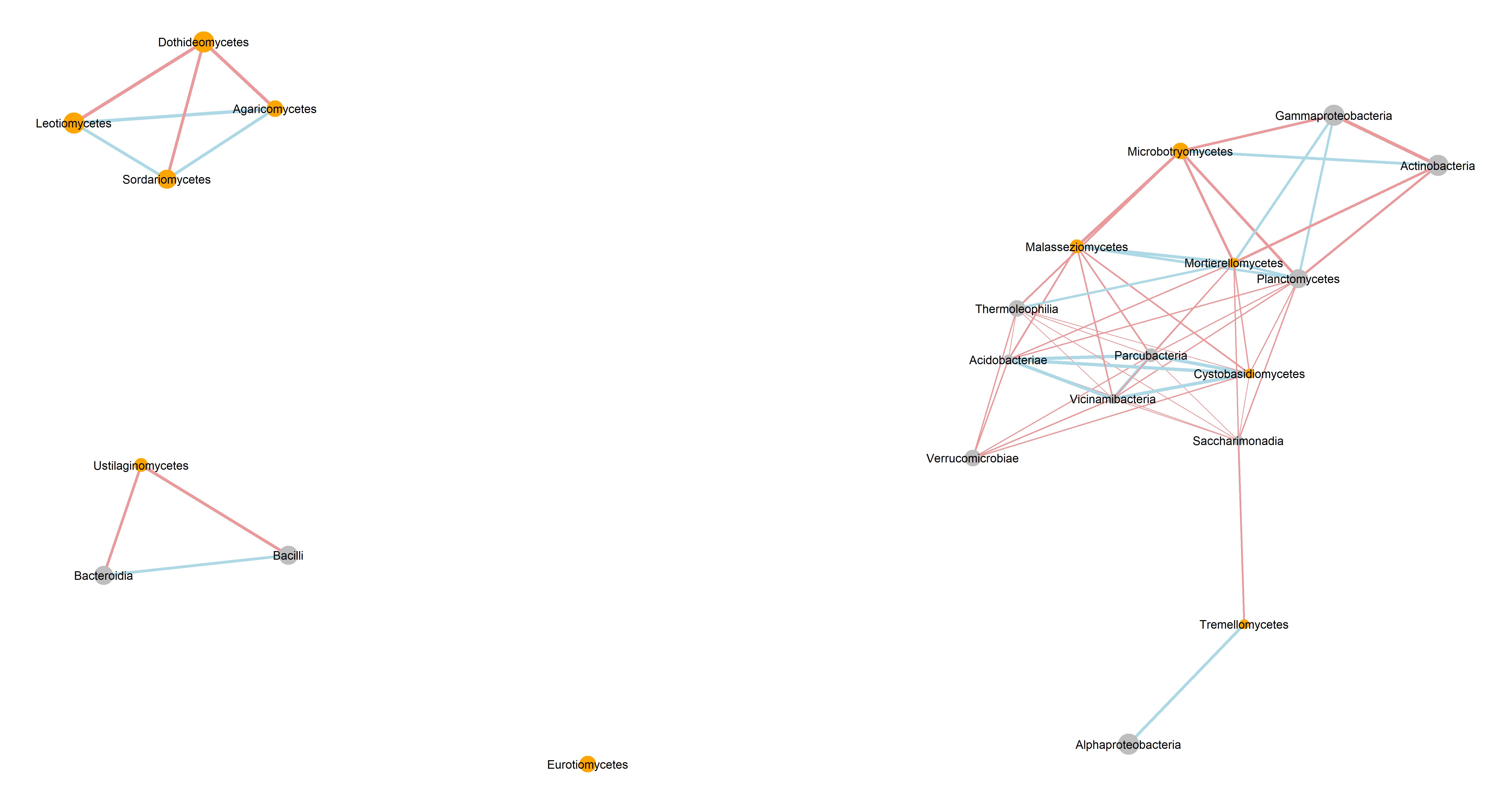

Supplement: Supplementary Figure 10 — Correlation network of wheat ears inoculated with S. rimosus subsp. rimosus LMG19352 at 14 days post inoculation at the taxonomical level of class. Edges represent positive (blue) or negative (red) correlations. The thickness of the edges is related to the absolute value of the correlation coefficient. Nodes represent fungal (orange) or bacterial (grey) dots. The size of each node is related to the relative abundance of the respective class (n = 5 biological replicates). [file Image10.jpeg]

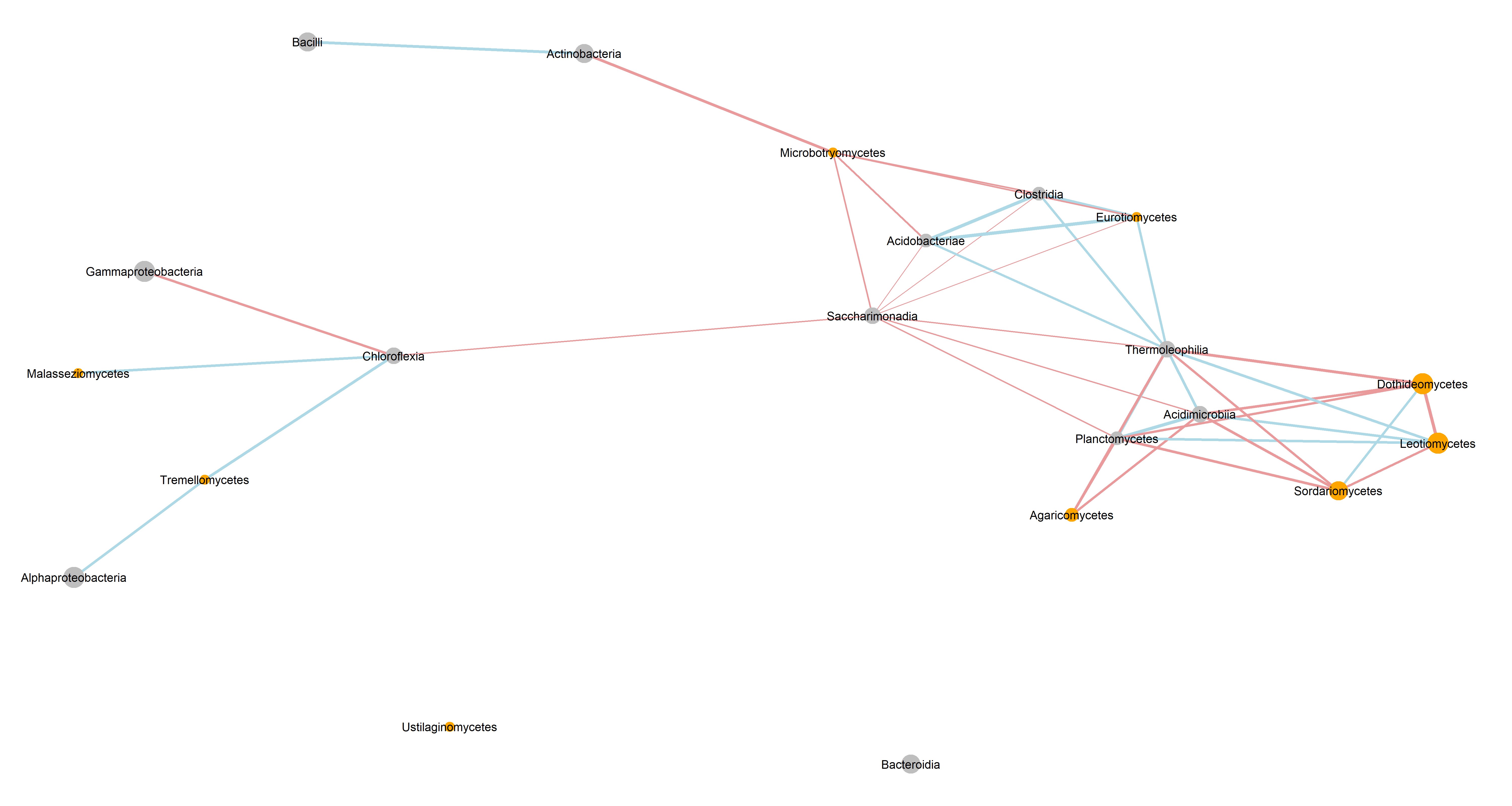

Supplement: Supplementary Figure 11 — Correlation network of wheat ears co-inoculated with S. rimosus subsp. rimosus LMG19352 and F. graminearum at 7 days post inoculation at the taxonomical level of class. Edges represent positive (blue) or negative (red) correlations. The thickness of the edges is related to the absolute value of the correlation coefficient. Nodes represent fungal (orange) or bacterial (grey) dots. The size of each node is related to the relative abundance of the respective class (n = 5 biological replicates). [file Image11.jpeg]

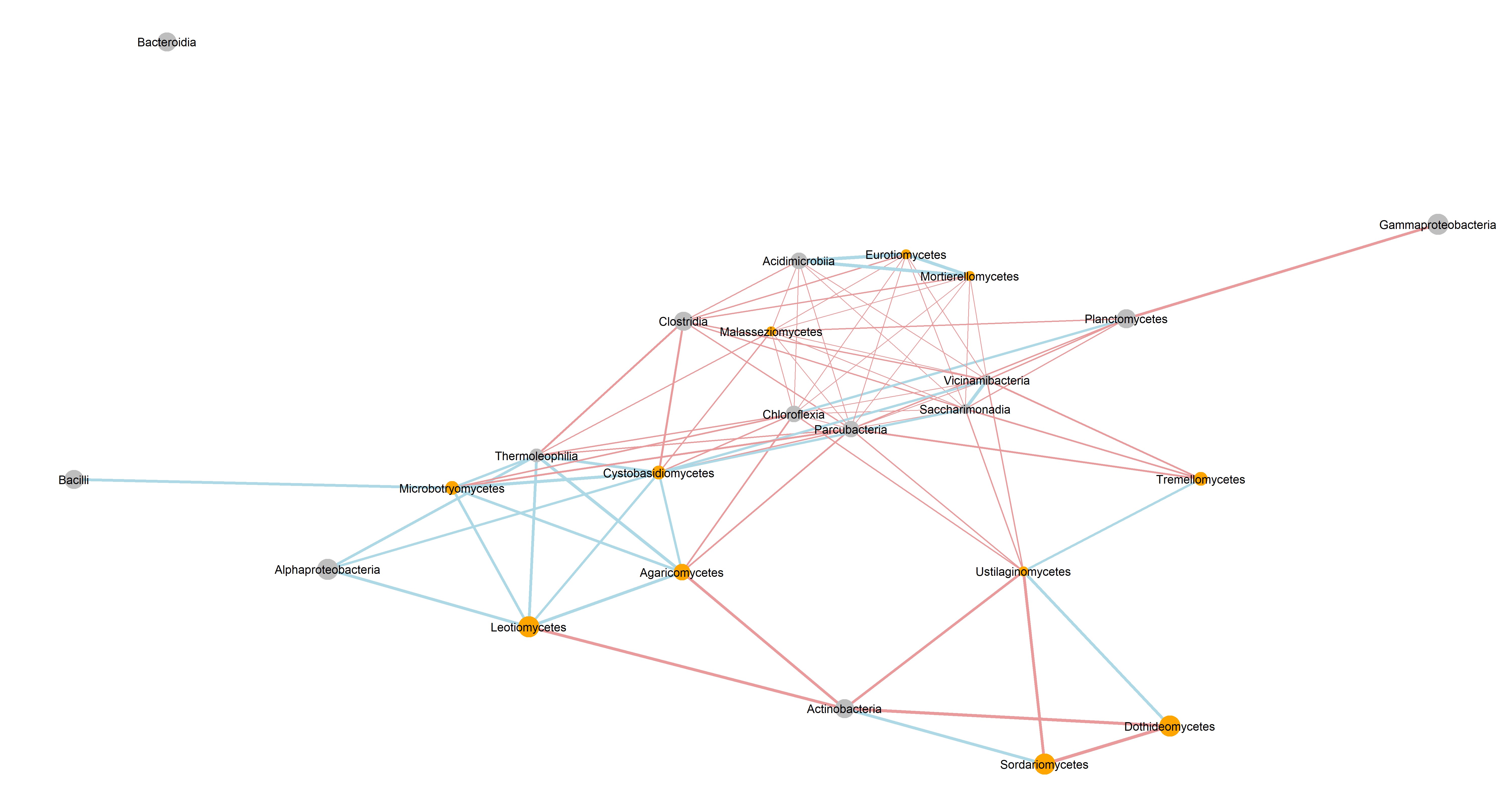

Supplement: Supplementary Figure 12 — Correlation network of wheat ears co-inoculated with S. rimosus subsp. rimosus LMG19352 and F. graminearum at 14 days post inoculation at the taxonomical level of class. Edges represent positive (blue) or negative (red) correlations. The thickness of the edges is related to the absolute value of the correlation coefficient. Nodes represent fungal (orange) or bacterial (grey) dots. The size of each node is related to the relative abundance of the respective class (n = 5 biological replicates). [file Image12.jpeg]
